# SERPINA4 facilitates colorectal cancer progression through m⁶A-dependent stabilization

**DOI:** 10.1186/s12967-026-07810-1

**Published:** 2026-02-28

**Authors:** Xiaoyu Zhang, Yutong Liu, Qianyi Tu, Dandan Zhang, Jiaqi Deng, Jingjiao Zhou, Hui Lv, Jun Zhou, Fengwu Qiu, Yang Deng

**Affiliations:** 1 Institute of Biology and Medicine, College of Life and Health Sciences, Wuhan University of Science and Technology, Wuhan, Hubei 430081 P.R. China; 2 Wuhan Blood Center, Wuhan, Hubei 430030 P.R. China; 3 College of Life Sciences, Jianghan University, Wuhan, Hubei 430081 P.R. China

**Keywords:** SERPINA4, Colorectal cancer, m6A, ALKBH5, IGF2BP3, Tumor microenvironment, Immune infiltration

## Abstract

**Background:**

Colorectal cancer (CRC) remains a leading cause of cancer-related mortality worldwide. Serine protease inhibitors (SERPINs) have emerged as potential regulators in tumor progression, yet the specific function and regulatory mechanisms of SERPINA4 in CRC remain poorly characterized.

**Methods:**

We integrated transcriptomic data from multiple CRC cohorts and conducted survival, pathway, and immune correlation analyses. Functional validation was performed using in vitro proliferation, migration, and invasion assays, as well as in vivo xenograft models. Post-transcriptional regulation of SERPINA4 via m6A modification was examined through MeRIP-qPCR, RNA immunoprecipitation, and decay assays.

**Results:**

High expression of SERPINA4 was significantly associated with poor prognosis across multiple survival endpoints. SERPINA4 overexpression promoted CRC cell growth and metastasis in vitro and enhanced tumor formation in vivo. Mechanistically, ALKBH5-mediated m⁶A demethylation reduced SERPINA4 transcript stability by weakening IGF2BP3 association. Rescue experiments with methylation-deficient mutants confirmed the necessity of m6A for SERPINA4-driven oncogenicity. Moreover, SERPINA4 expression correlated with immunosuppressive signatures and reduced CD8 + T cell infiltration.

**Conclusions:**

Our findings reveal that SERPINA4 facilitates CRC progression through both tumor-intrinsic mechanisms and modulation of the immune microenvironment. These findings support an epitranscriptomic regulatory mechanism involving an ALKBH5–m6A–IGF2BP3 axis in CRC. SERPINA4 may serve as a prognostic biomarker and therapeutic target in precision oncology.

**Supplementary Information:**

The online version contains supplementary material available at 10.1186/s12967-026-07810-1.

## Introduction

Colorectal cancer is a common malignant tumor of the digestive system, characterized primarily by uncontrolled proliferation and sustained survival of abnormal cells within the colon or rectum. As the disease progresses to advanced stages, the tumor can metastasize to other normal tissues or organs, leading to high occurrence rates and mortality [1, 2]. In 2022, deaths attributable to CRC accounted for nearly 10% of all cancer-related deaths. Predictions indicate that the disease burden associated with CRC will increase by approximately 63% by 2040 [3]. Although treatment strategies for CRC have improved in recent years, the overall therapeutic efficacy remains suboptimal, particularly for patients with advanced disease, due to the frequent development of drug resistance to non-surgical strategies such as radiotherapy, chemotherapy, targeted therapy, and immunotherapy [4]. Furthermore, early diagnosis is crucial for improving patient survival rates. However, owing to the complex biology of CRC and the lack of molecular markers with high sensitivity and specificity, current early screening still relies predominantly on invasive examinations such as endoscopy [5]. Therefore, the discovery of novel biomarkers holds significant clinical value for the early precise diagnosis and prognostic assessment of CRC [6].

Serine protease inhibitors (Serpins) are a class of structurally similar and functionally diverse plasma proteins. Serpin Family A Member 4 (SERPINA4) belongs to this protein class and was first identified in the 1980s as a human serum tissue kallikrein-binding protein. Its structure comprises two main functional regions: a heparin-binding pocket and an active site [7]. Studies have shown that SERPINA4 can inhibit angiogenesis and inflammatory responses, and induce apoptosis in cultured human endothelial cells, by suppressing TNF-α-mediated activation of the NF-κB pathway and blocking the VEGF signaling pathway [8–10]. Additionally, SERPINA4 has been reported to inhibit the proliferation, migration, and invasion of various malignancies, including lung cancer, breast cancer, and hepatocellular carcinoma, by modulating cancer cell signal transduction [11–14]. Notably, dysregulated SERPINA4 expression in CRC has been reported to be associated with tumor aggressiveness and clinical outcomes [6]. However, while SERPINA4 is a canonical serpin and its protease-inhibitory activity may plausibly influence invasion-related processes, whether this inhibitory function is mechanistically required for CRC progression has not been directly tested in the present study and remains to be determined.

N6-methyladenosine (m6A) modification is one of the most prevalent chemical modifications of mRNA in mammalian cells, significantly influencing mRNA stability, splicing, nuclear export, subcellular localization, and translation efficiency [15]. m6A modification is dynamic and reversible, regulated by the coordinated actions of methyltransferase complexes (“writers”), demethylases (“erasers”), and m6A-binding proteins (“readers”) [16]. Recent studies indicate that aberrant m6A modification is closely associated with tumor metabolism [17–19]. In CRC cells, upregulating FTO reduces the m6A modification level of Activating Transcription Factor 4 (ATF4) mRNA, thereby promoting ATF4 expression. Subsequently, increased ATF4 further elevates the level of DNA Damage Inducible Transcript 4 (DDIT4), leading to inactivation of the mTOR pathway under glutaminolysis inhibition and promoting pro-survival autophagy [20].

ALKBH5 is a key m6A demethylase belonging to the non-heme Fe(II)/2-OG-dependent dioxygenase family, which catalyzes the oxidation of various substrates including nucleic acids, lipids, proteins, and small molecule metabolites [21]. As an m6A demethylase, ALKBH5 exhibits a diverse demethylation profile, with RNA substrate preferences and tissue-specific expression patterns [22]. Notably, ALKBH5 is primarily localized in nuclear speckles and can directly catalyze the demethylation of m6A to adenosine without intermediates, displaying unique structural characteristics [23, 24]. For instance, in human hepatocellular carcinoma (HCC) tissues and Huh-7 cells, ALKBH5 is significantly downregulated and acts as a tumor suppressor by regulating glycolysis and inhibiting tumor growth [25]. Furthermore, ALKBH5 is involved in the anticancer process related to glutathione depletion induced by the curcumin analog WZ35, via the ROS-YAP-AXL-GLS2 signaling axis [26]. Accumulating evidence demonstrates that ALKBH5 is closely involved in the development and progression of various malignancies, with roles in promoting tumor cell proliferation, invasion, and metastasis, while also modulating therapeutic responses, cancer stem cell (CSC) characteristics, the tumor microenvironment (TME), and immune responses [27, 28].

## Materials and methods

### Expression and prognostic value analysis of key SERPIN family genes

A prognostic signature was first derived from the TCGA cohort using Lasso regression. To assess whether SERPIN family members serve as independent prognostic factors, multivariate Cox regression was then performed with the survival (v3.2.10; http://CRAN.R-project.org/package=survival) and rms (v6.3-0; http://hbiostat.org/R/rms/) packages. The resulting regression coefficients (β) were combined with corresponding gene expression profiles to establish a multi-gene risk scoring system, which was trained on TCGA data. Calculation of individual risk scores was conducted using the glmnet package (v4.1.7).

In both the TCGA COAD cohort (*n* = 461) and the GSE14333 CRC cohort (*n* = 290), patients were divided into high- and low-risk subgroups according to the median score. Survival outcomes were compared between groups using Kaplan–Meier analysis and two-sided log-rank testing, with statistical significance defined as *P* < 0.05. The predictive accuracy of the model was subsequently evaluated through time-dependent ROC curve analysis.

### Identification of hub genes

The 35 SERPIN family members were uploaded to the STRING database (https://cn.string-db.org/) to perform protein–protein interaction (PPI) analysis. Candidate hub genes within modular subnetworks were subsequently detected using Cytoscape software (v3.9.1) [29] combined with the CytoHubba plugin [30]. Additional validation was conducted through clustering coefficient–based methods.

### Pan-cancer gene expression and immune infiltration analysis

To characterize the expression landscape of key SERPIN family members across multiple cancer types, a pan-cancer analysis was performed using the Xiantao platform (https://www.xiantao.love/). Gene expression levels in tumor samples were contrasted with those in corresponding adjacent normal tissues. Furthermore, associations between SERPIN expression and the infiltration of immune cell subsets—such as T cells, B cells, and dendritic cells—were systematically evaluated.

### Survival analysis

Kaplan–Meier survival analyses were carried out using the survival (v3.2.10) and survminer (v0.4.9; http://CRAN.R-project.org/package=survminer) packages. After excluding normal tissues and samples lacking clinical data, TCGA-COAD patients were grouped according to SERPINA4 expression levels to assess prognostic value in overall survival (OS, *n* = 1061), relapse-free survival (RFS, *n* = 1336), progression-free interval (PFI, *n* = 521), and disease-specific survival (DSS, *n* = 521). The diagnostic capability of SERPINA4 was further evaluated through ROC curve analysis using the pROC package (v1.18.0; http://rdocumentation.org/packages/pROC/versions/1.18.5). In addition, Cox regression models were fitted with the survival package, and forest plots were generated in ggplot2 (v3.3.3; http://ggplot2.tidyverse.org).

### Functional enrichment analysis

Spearman correlation analysis of SERPINA4 was conducted using the LinkedOmics database (http://www.linkedomics.org/login.php) [31], applying a threshold of *P* < 0.01, which identified 10,369 associated genes. Genes with correlation coefficients |r|>0.3 were retained for further investigation. Gene functional enrichment, including Gene Ontology (GO) and Kyoto Encyclopedia of Genes and Genomes (KEGG) analyses, was performed using clusterProfiler (v4.4.4) [32], and results were visualized with ggplot2. In parallel, gene set enrichment analysis (GSEA) was performed on TCGA COAD data via the Xiantao Academic platform with curated pathway gene sets from Reactome and WikiPathways (as indicated by the pathway source labels in the figures), using cutoffs of *P* < 0.01 and |log₂FC|>2, and pathway Z-scores were calculated.

### Immunological analysis

Single-sample Gene Set Enrichment Analysis (ssGSEA) was applied using GSVA (v1.46.0) [33] to assess the relationship between SERPINA4 expression and the infiltration characteristics of 24 distinct immune cell subsets. It should be noted that ssGSEA provides transcriptome-based estimates of immune cell infiltration and does not directly quantify tissue-level immune cell abundance. Additionally, correlations between SERPINA4 and a range of immune-related factors—including chemokines, inhibitory and stimulatory molecules, MHC components, and immune receptors—were investigated using the TISIDB database (http://cis.hku.hk/TISIDB/) [34].

### In vitro functional experiments

Wound healing and Transwell migration assays were conducted as previously described [35] with minor modifications. For the wound healing assay, RKO cells were grown in standard culture medium until fully adherent, then maintained in 1% serum medium for 48 h to evaluate migratory capacity. In the Transwell assay, RKO cells were seeded into the upper chamber and cultured for 48 h to assess invasive potential.

### CCK-8

Cell proliferation was assessed using the CCK-8 assay. Cells were plated in 96-well plates, and CCK-8 reagent (Dalian Meilun Biotech Co., China) was added at predetermined time points. After a 2-hour incubation, absorbance at 450 nm (OD450) was measured to determine cell viability.

### Western blot

Western blotting was carried out as previously described [35]. Primary antibodies included SERPINA4 (PA5-31437, Thermo Fisher, China; 1:2000), GAPDH (60004-1-Ig, Proteintech, Wuhan, China; 1:50000), MMP2 (10373-2-AP, Proteintech, Wuhan, China; 1:2000), and VIM (10366-1-AP, Proteintech, Wuhan, China; 1:2000).

### RT-qPCR

RT-qPCR experiments followed the method in reference [35]. Primer sequences were as follows:

SERPINA4-F: 5′-CCTGCTGGTTGGACTACTGG-3′, R: 5′-CTGTTACTGCAACTCTCACCAT-3′.

ALKBH5-F: 5′-CGGCGAAGGCTACACTTACG-3′, R: 5′-CCACCAGCTTTTGGATCACCA-3′.

GAPDH-F: 5′-TGCACCACCAACTGCTTAGC-3′, R: 5′-GGCATGGACTGTGGTCATGAG-3′.

### Cell lines and culture

NCM460, SW480, and RKO cell lines were purchased from Cyagen Biosciences (Shanghai, China) and authenticated by the supplier. Cells were maintained in DMEM supplemented with 10% fetal bovine serum (Hyclone) and 1% penicillin–streptomycin at 37 ℃ in a humidified incubator with 5% CO_2_. Culture medium was replaced every 2–3 days, and cells were subcultured with 0.25% trypsin–EDTA once they reached 80–90% confluence.

### Stable cell line construction

HEK-293T cells in the logarithmic growth phase (5 × 10⁶ cells/mL) were transfected with SERPINA4-targeting shRNA lentiviral plasmids (Qink Biotech) and packaging plasmid systems using Lipofectamine™ 2000 (Invitrogen, USA) to generate lentiviral particles. After 24 h, the medium was replaced, and viral supernatants were collected 72 h post-transfection, filtered through a 0.45 μm membrane, and stored at − 80 ℃. Viral titers were quantified with the Lenti-Pac™ HIV RT-qPCR Titration Kit (GeneCopoeia, USA). RKO cells were subsequently infected and selected with puromycin (2 µg/mL) to establish stable knockdown lines. The shRNA sequence used was: sh-SERPINA4, 5′-CCCTTCCTTGTGGTGATCTTT-3′. For overexpression models, RKO cells were transfected with SERPINA4 expression plasmids (Beijing Kewen Biotech, China) via Lipofectamine™ 2000, and positive clones were selected following the same procedure.

### Nude mouse xenograft model

Xenograft experiments followed the method described in reference [36]. The mice in the oe-NC and oe-SERPINA4 groups were euthanized 12 days after injection, whereas those in the sh-NC and sh-SERPINA4 groups were euthanized 17 days after injection.

### Immunohistochemistry

Mouse subcutaneous tumor tissues were fixed, embedded, and sectioned by Fabritus Biotech (Wuhan, China), followed by staining according to their standardized immunohistochemistry protocols.

### ActD assay

Cells were plated in 6-well plates at a density of 5 × 10⁵ cells per well and cultured for 24 h until reaching 70–80% confluence. The medium was subsequently replaced with fresh medium containing 5 µg/mL ActD (MEI5BIO, #A67895). At 0, 2, 4, 8, and 12 h, cells were collected, and total RNA was isolated for RT-qPCR analysis to evaluate mRNA degradation kinetics and estimate transcript half-lives.

### RIP assay

RNA immunoprecipitation was performed with the Magna RIP™ Kit (Millipore, #17–700). Cell lysates were incubated with either an anti-IGF2BP3 antibody (Abcam, ab177477) or IgG-conjugated control magnetic beads. The captured RNA was subsequently purified and subjected to RT-qPCR to evaluate the enrichment of target transcripts.

### MeRIP assay

RNA methylation immunoprecipitation was carried out using the Magna MeRIP™ m6A Kit (Millipore, #17-10499). Fragmented RNA was incubated with anti-m6A antibody (Synaptic Systems, #202 003) bound to magnetic beads. The enriched RNA was then purified and quantified by RT-qPCR to evaluate m6A levels on specific transcripts, expressed as a percentage of input.

### m6A site prediction and SERPINA4 mutant design

**Fig. 1:**
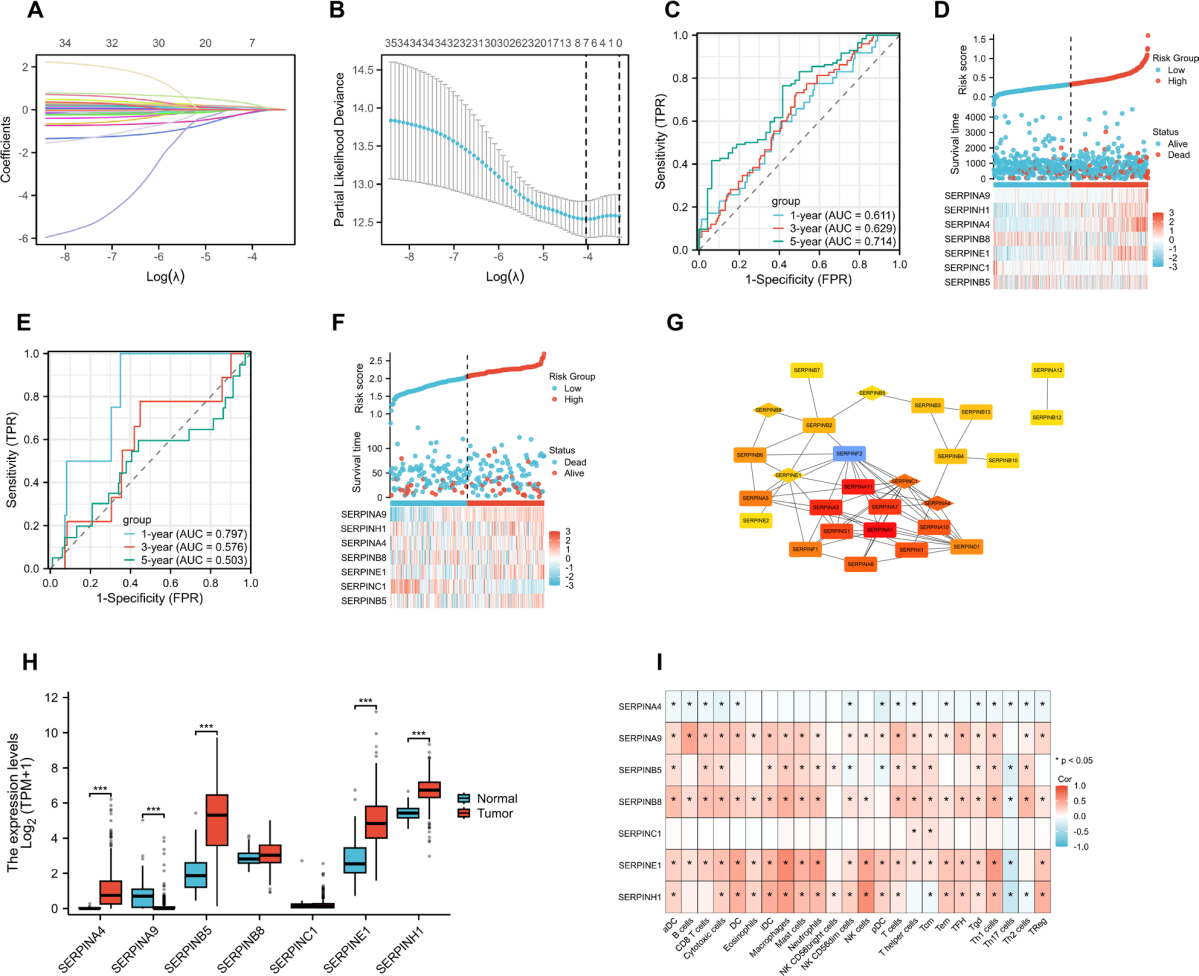
Overview of the SERPIN gene family in colorectal cancer. (**A**) LASSO coefficient distribution of candidate genes. (**B**) Cross-validation of parameter selection in the proportional hazards model. (**C**) Time-dependent ROC curve for 1-year, 3-year, and 5-year survival rates. (**D**) Distribution of risk scores, survival status, and gene expression profiles of the seven genes in the TCGA dataset. (**E**-**F**) External validation results from the GSE14333 dataset. (**G**) Protein-protein interaction network of prognostic-related SERPIN genes. (**H**) Comparison of expression differences between tumor and normal tissues. (**I**) Correlation analysis of SERPIN expression and immune cell infiltration. *, *P* < 0.05; **, *P* < 0.01, ***, *P* < 0.001

Putative m6A modification sites on SERPINA4 mRNA were predicted using the SRAMP online tool. Based on the prediction results, the candidate site around position 695 showed the highest confidence/combined score and was therefore prioritized for mutant construction. The methylation-deficient SERPINA4 mutant (mut695) was generated by site-directed mutagenesis to disrupt the predicted m6A consensus motif, and WT and mut695 constructs were used for the rescue experiments described in Fig. 1.

### Statistical analysis

All statistical analyses were performed using GraphPad Prism (v8.0.1; Dotmatics). Unless otherwise stated, experiments were independently repeated at least three times. Data are presented as mean ± SD for in vitro assays and as mean ± SEM for tumor growth curves, as indicated in the corresponding figure legends. For comparisons between two groups, unpaired two-tailed Student’s t-test was used. For comparisons among three or more groups, one-way ANOVA followed by Tukey’s multiple-comparison test was applied. A P value < 0.05 was considered statistically significant. Statistical significance is indicated by *P < 0.05, **P < 0.01, and ***P < 0.001, as shown in the figure legends. All experiments were independently repeated at least three times to ensure the reliability of the results.

## Results

### Prognostic and immunological association of the SERPIN gene family in colorectal cancer

To investigate the prognostic relevance of SERPIN family members, key SERPIN genes were identified from the TCGA-COAD cohort using LASSO regression. Seven risk-associated candidates—SERPINA9, SERPINH1, SERPINA4, SERPINB8, SERPINE1, SERPINC1, and SERPINB5—were selected (Fig. 1A–B). ROC analysis confirmed the predictive capacity of the model, with notable performance at 5-year follow-up (AUC = 0.714; Fig. 1C). Patients were stratified into high- and low-risk groups according to the median risk score derived from SERPIN gene expression. As shown in Fig. 1D, survival time, survival status, and gene expression distribution indicated poorer prognosis and higher mortality in the high-risk group.Validation in the independent GSE14333 dataset demonstrated consistent risk score patterns and ROC performance (Fig. 1E–F), confirming the robustness of the prognostic model. Functional associations among the seven SERPIN genes were further illustrated by the PPI network (Fig. 1G). Comparative expression analysis revealed significant upregulation of several SERPINs in tumor samples relative to adjacent normal tissues (Fig. 1H). Additionally, correlation analysis highlighted strong associations between SERPIN expression and immune cell infiltration, including T cells, B cells, and dendritic cells, suggesting their involvement in modulating the tumor immune microenvironment (Fig. 1I).Among the seven candidates, SERPINA4 showed the most pronounced differential expression between TCGA high- and low-risk groups, with a relatively higher coefficient and risk weight in the LASSO model. Although previous studies have reported SERPINA4 as an independent prognostic factor in CRC [6], its molecular regulatory mechanisms and functional roles remain insufficiently characterized. Therefore, SERPINA4 was prioritized for further investigation in subsequent bioinformatics analyses.

### Impact of SERPINA4 expression on colorectal cancer prognosis

**Fig. 2:**
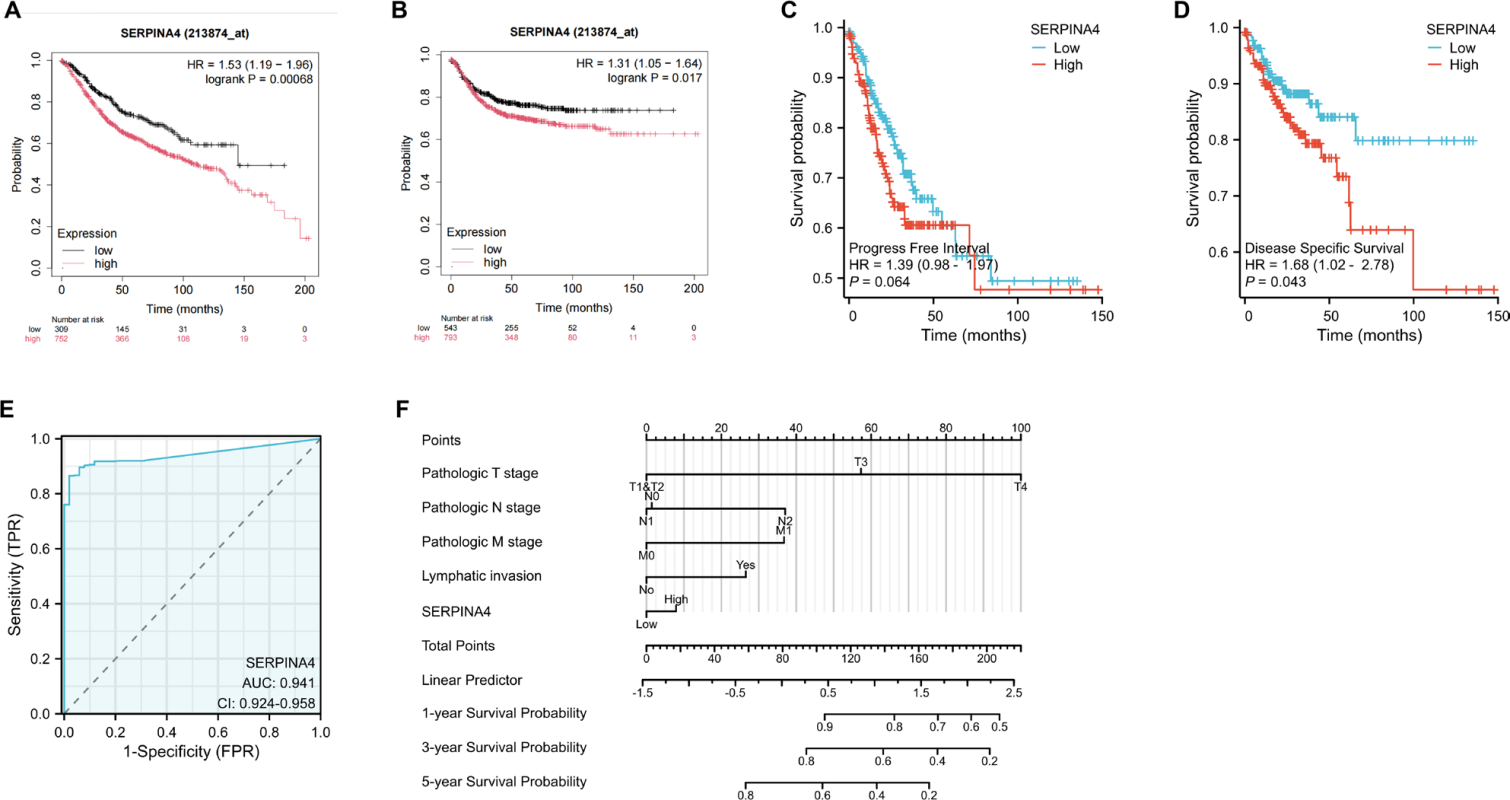
Prognostic value of SERPINA4 in colorectal cancer. (**A**-**D**) Kaplan-Meier survival curves for OS, RFS, PFI, and DSS stratified by SERPINA4 expression levels. (**E**) ROC curve evaluation of the survival prediction model. (**F**) Nomogram for survival analysis combining clinical features and SERPINA4 expression. Log-rank test used for survival comparisons; P-value < 0.05 indicates statistical significance

To further clarify the role of SERPINA4, its expression was examined across multiple survival endpoints. Kaplan–Meier analyses revealed that patients with elevated SERPINA4 expression exhibited significantly shorter overall survival (OS), relapse-free survival (RFS), and disease-specific survival (DSS), as well as a reduced progression-free interval (PFI), indicating an association with more aggressive disease behavior (Fig. 2A–D).ROC curve analysis based on TCGA colorectal cancer data demonstrated excellent diagnostic performance of SERPINA4, with an AUC of 0.941 (95% CI: 0.924–0.958), confirming its strong discriminative power between cancer and normal states (Fig. 2E). Moreover, integration of SERPINA4 expression with clinical variables, including pathological stage and lymph node metastasis, enabled the construction of a robust prognostic model capable of predicting 1-, 3-, and 5-year survival probabilities (Fig. 2F).

### Functional enrichment analysis of SERPINA4-related pathways

**Fig. 3:**
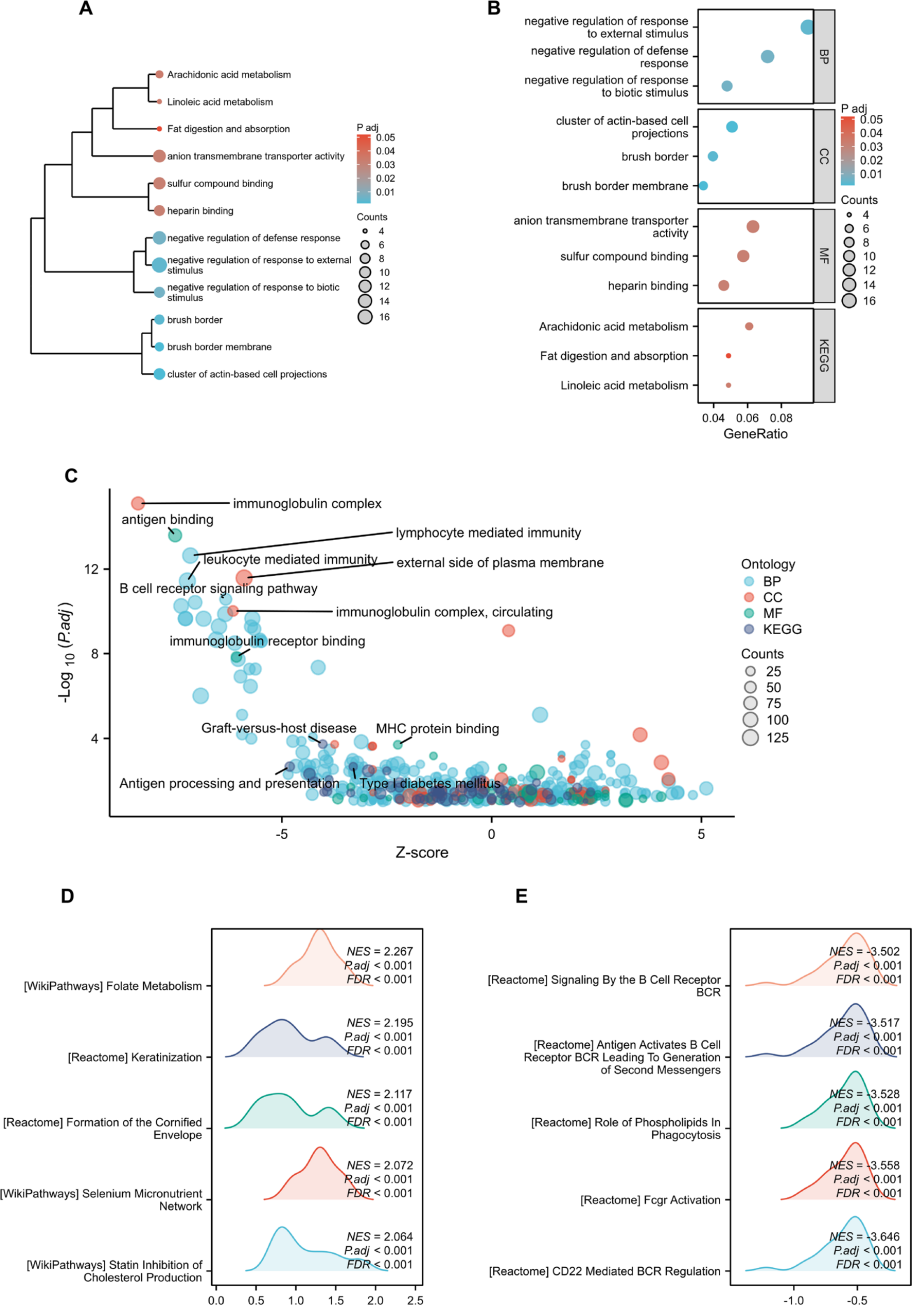
Pathway enrichment analysis related to SERPINA4 expression. (**A**) Clustering dendrogram of enriched pathways based on TCGA-COAD samples stratified by SERPINA4 expression. (**B**) Gene ratio plot for the top-ranked pathways. (**C**) Bubble plot of GO and KEGG terms. (**D**–**E**) GSEA plots showing representative immune/metabolic pathways enriched in the high-SERPINA4 group. GSEA was performed using the MSigDB gene set collection specified in the Methods; normalized enrichment score (NES) and FDR q-values are indicated in each plot

GSEA was performed to investigate the potential biological functions of SERPINA4. The analysis revealed significant enrichment of metabolism-related pathways, including folate biosynthesis, arachidonic acid metabolism, and lipid absorption. These metabolic processes are closely linked to immune activity, suggesting a multifaceted role of SERPINA4 in tumor progression. The clustering dendrogram and gene ratio plot illustrated hierarchical interactions between immune and metabolic pathways, supporting the notion that SERPINA4 contributes to both immune regulation and metabolic reprogramming in cancer (Fig. 3A–B).Furthermore, immune-associated pathways such as B cell receptor signaling and immunoglobulin complex formation were predominantly enriched in the high-expression group (Fig. 3C–E). Collectively, these findings suggest an association between SERPINA4 expression and immune-related pathways within colorectal tumors.

### Correlation of SERPINA4 with immune-related gene signatures

**Fig. 4:**
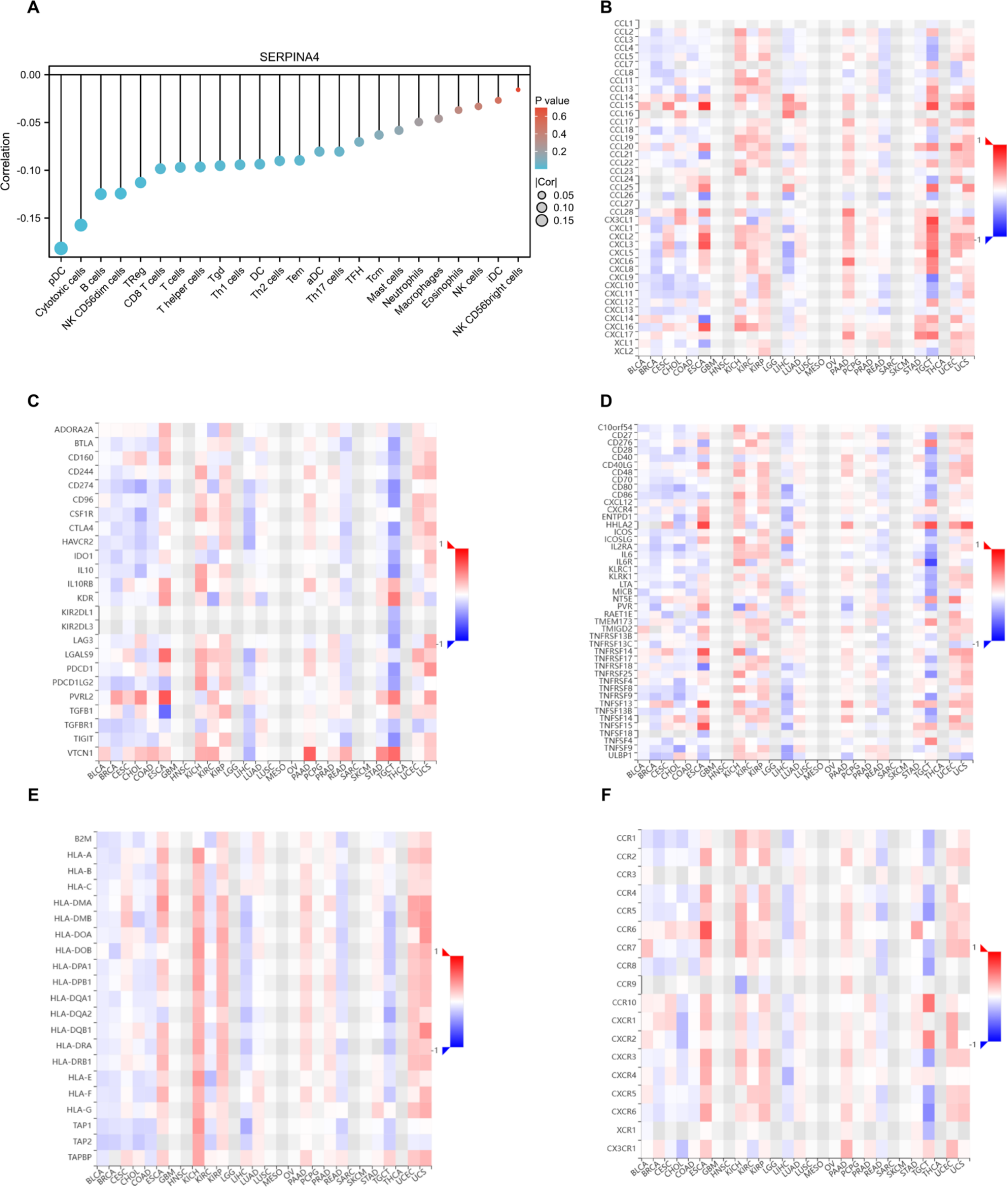
Correlation of SERPINA4 expression with immune gene signatures. (**A**) Relationship with CD8 + T and NK cells. (**B**) Association with chemokines (CXCL and CCL families). (**C**) Correlation with immune checkpoint markers (PD-1, CTLA4). (**D**) Linkage with immune stimulatory genes (CD28, CD40L). (**E**-**F**) Correlation with MHC and antigen receptor genes (TCR, BCR). Spearman correlation; significance correction for multiple comparisons

Comprehensive analysis revealed that SERPINA4 expression is significantly correlated with multiple immune regulatory genes. Notably, its expression showed negative correlations with CD8⁺ T cells and NK cells, suggesting a potential association with immune-related features (Fig. 4A). These associations are based on computational inference from bulk transcriptomic data and warrant further tissue-level validation. In addition, SERPINA4 displayed both positive and negative associations with chemokine families such as CXCL and CCL, indicating a possible correlation with chemokine-related immune signaling (Fig. 4B). SERPINA4 expression was also positively correlated with immune checkpoint molecules, including PD-1 and CTLA4, as well as immune stimulatory factors such as CD28 and CD40L (Fig. 4C–D). Moreover, correlations were observed with genes encoding MHC class I/II molecules and antigen receptor components such as TCR and BCR (Fig. 4E–F). Together, these findings suggest that SERPINA4 expression is associated with multiple immune-regulatory signatures, which may reflect heterogeneity of the immune context across CRC cohorts.

### SERPINA4 enhances colorectal cancer cell proliferation, migration, and invasion in vitro

**Fig. 5:**
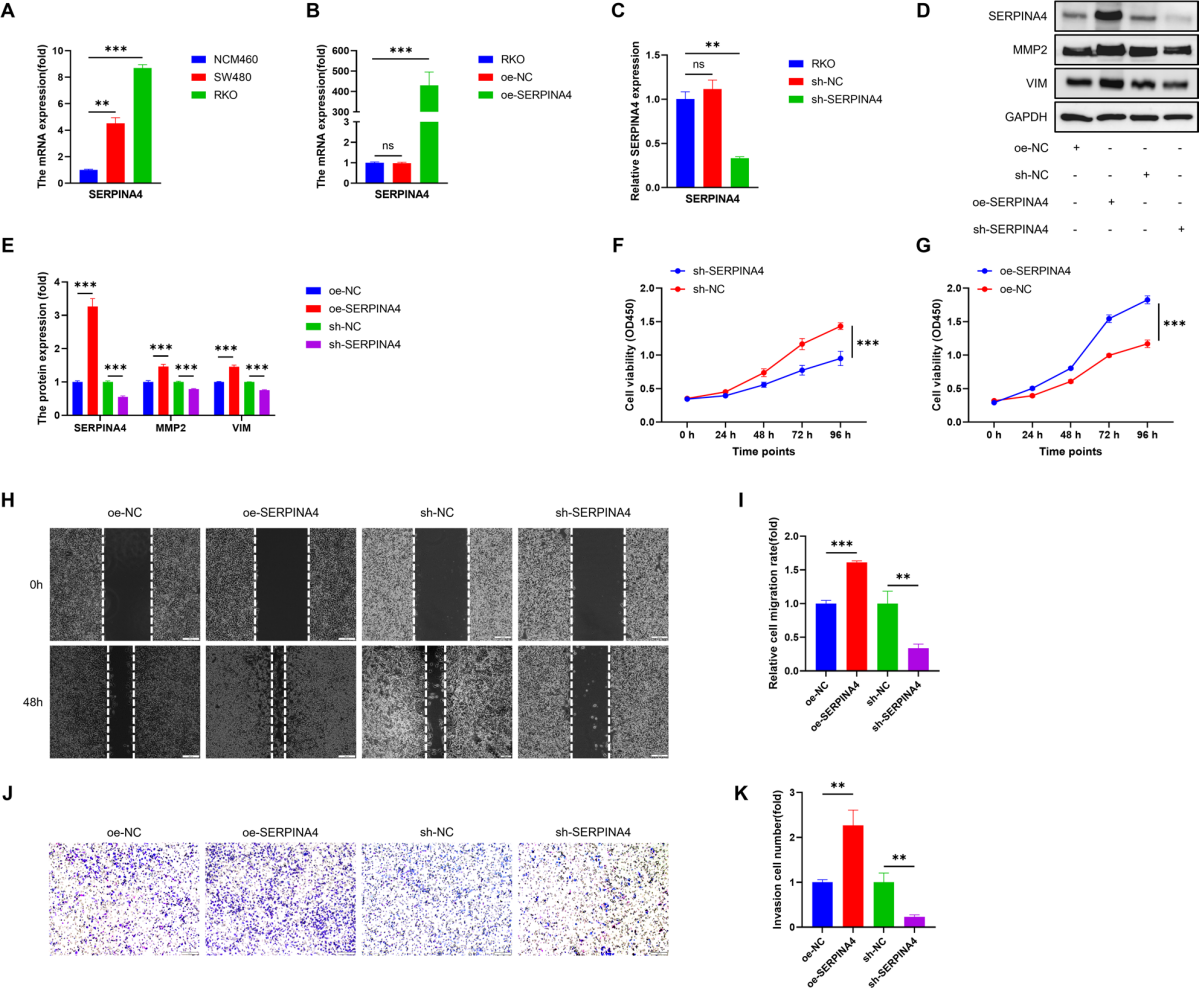
SERPINA4 promotes CRC cell proliferation and motility in vitro. (**A**) Baseline SERPINA4 expression in CRC cell lines. (**B**–**C**) RT-qPCR validation of overexpression and knockdown. (**D**–**E**) Western blot for MMP2 and VIM. (**F**–**G**) Cell proliferation by CCK-8 assay. (**H**–**I**) Wound healing migration analysis. (**J**–**K**) Transwell migration and invasion assays. Data are presented as mean ± SD from at least three independent experiments. Student’s t-test used for comparison. *, *P* < 0.05; **, *P* < 0.01, ***, *P* < 0.001

To further validate the transcriptional upregulation of SERPINA4 in colorectal cancer, we assessed its mRNA expression levels in CRC cell lines and a normal colonic epithelial cell line. As shown in Fig. 5A, SERPINA4 expression was significantly elevated in SW480 and RKO cells compared with the normal NCM460 cells. Subsequently, RKO cell lines of oe-SERPINA4 as well as sh-SERPINA4 were constructed, and changes in their mRNA expression levels were detected by RT-qPCR (Fig. 5B–C). Given the enhanced motility observed in functional assays, we next examined EMT-associated mesenchymal markers. Western blotting showed that SERPINA4 overexpression was associated with increased MMP2 and VIM levels, whereas SERPINA4 knockdown reduced their expression (Fig. 5D–E). Proliferation assays (CCK-8) confirmed that SERPINA4 overexpression markedly enhanced cell growth, whereas silencing of SERPINA4 reduced it (Fig. 5F–G).

**Fig. 6:**
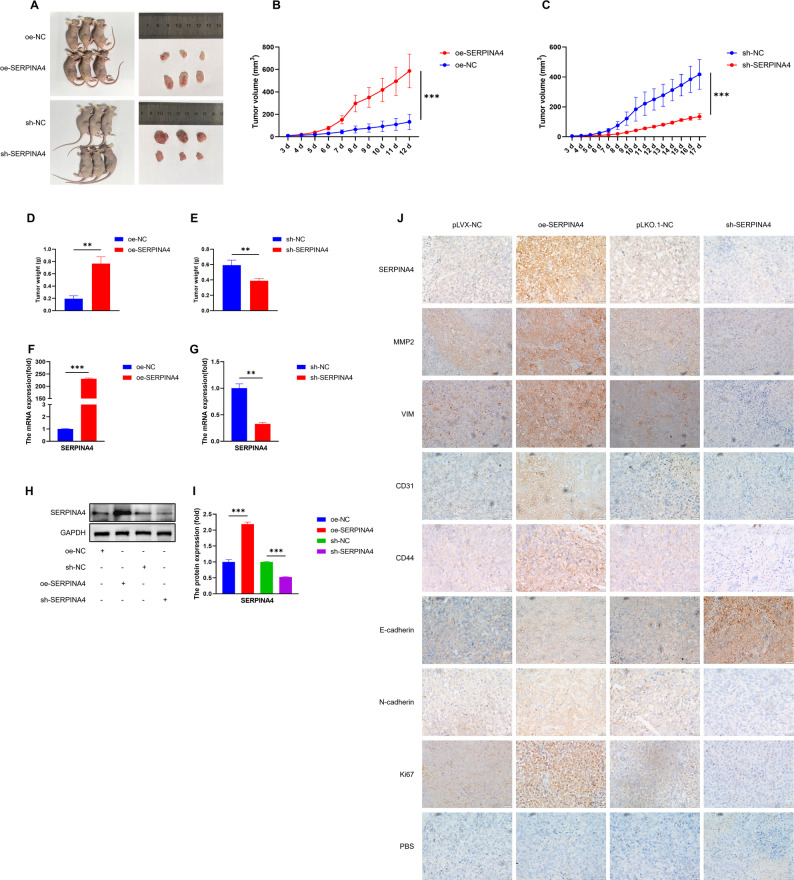
SERPINA4 enhances tumor growth in a xenograft model. (**A**–**C**) Tumor images and volume curves from nude mice. (**D**–**E**) Final tumor weights. (**F**–**G**) RT-qPCR for SERPINA4 expression in tumor tissues. (**H**–**I**) Western blot for SERPINA4 protein. (**J**) IHC staining of MMP2, VIM, CD31, CD44, Ki67, and EMT markers. Data are presented as mean ± SEM (tumor growth curves) or mean ± SD (endpoint measurements), as indicated. Statistical significance was assessed using unpaired two-tailed Student’s t-test. *n* = 3 mice per group. *, *P* < 0.05; **, *P* < 0.01; ***, *P* < 0.001

Moreover, scratch wound healing and transwell assays revealed that SERPINA4 overexpression promoted both migratory and invasive capabilities, whereas knockdown impaired these processes (Fig. 6H–K).

### SERPINA4 promotes tumor growth in vivo in a xenograft mouse model

To evaluate the in vivo effects of SERPINA4, a xenograft model was established by subcutaneously injecting RKO cells into nude mice. Tumors derived from SERPINA4-overexpressing cells displayed significantly accelerated growth, while tumors with SERPINA4 knockdown exhibited slower progression and smaller volumes (Fig. 6A–C). Consistently, tumor weights measured at the endpoint were notably higher in the oe-SERPINA4 group and lower in the sh-SERPINA4 group (Fig. 6D–E). RT-qPCR and western blot analyses confirmed higher mRNA and protein expression of SERPINA4 in the overexpression group and suppressed levels in the knockdown group (Fig. 6F–I). Immunohistochemical staining of tumor tissues revealed that overexpression of SERPINA4 enhanced the expression of invasion- and angiogenesis-related markers, including MMP2, VIM, CD31, CD44, and Ki67, while downregulation reversed these changes (Fig. 6J).

### ALKBH5 regulates SERPINA4 expression through m⁶A modification and IGF2BP3-dependent post-transcriptional regulation

**Fig. 7:**
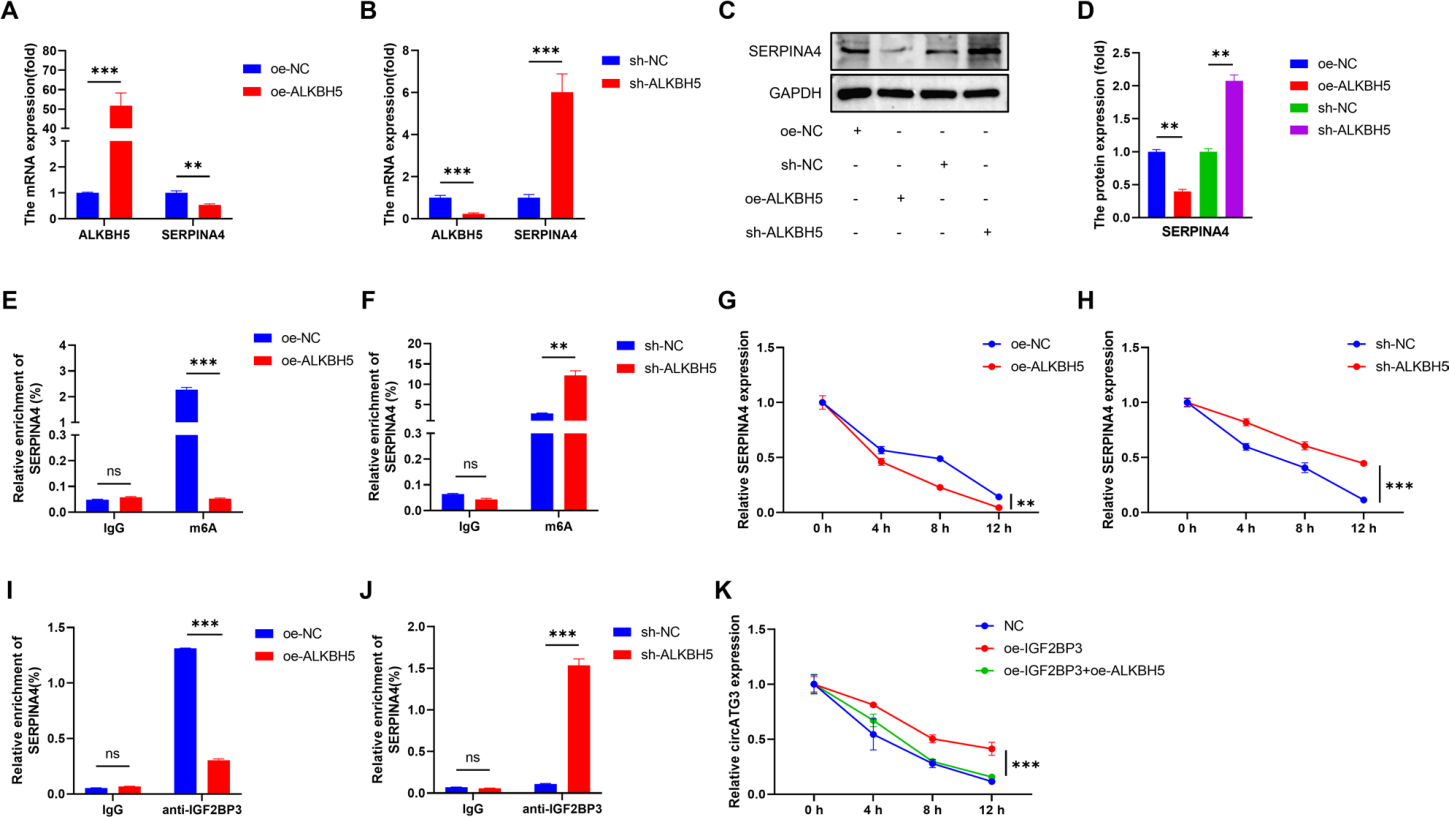
Regulation of SERPINA4 by ALKBH5 via m6A and IGF2BP3. (**A**–**B**) SERPINA4 mRNA levels upon ALKBH5 modulation. (**C**–**D**) Protein levels of SERPINA4 after ALKBH5 overexpression/knockdown. (**E**–**F**) MeRIP-qPCR showing m⁶A enrichment on SERPINA4 transcripts upon ALKBH5 overexpression or knockdown. (**G**–**H**) mRNA decay curves using Actinomycin D. (**I**–**J**) RIP assays demonstrating SERPINA4–IGF2BP3 binding. (**K**) Stability assay with IGF2BP3 overexpression. Values represent mean ± SD from triplicate experiments. Statistical significance by Student’s t-test. *, *P* < 0.05; **, *P* < 0.01, ***, *P* < 0.001

**Fig. 8:**
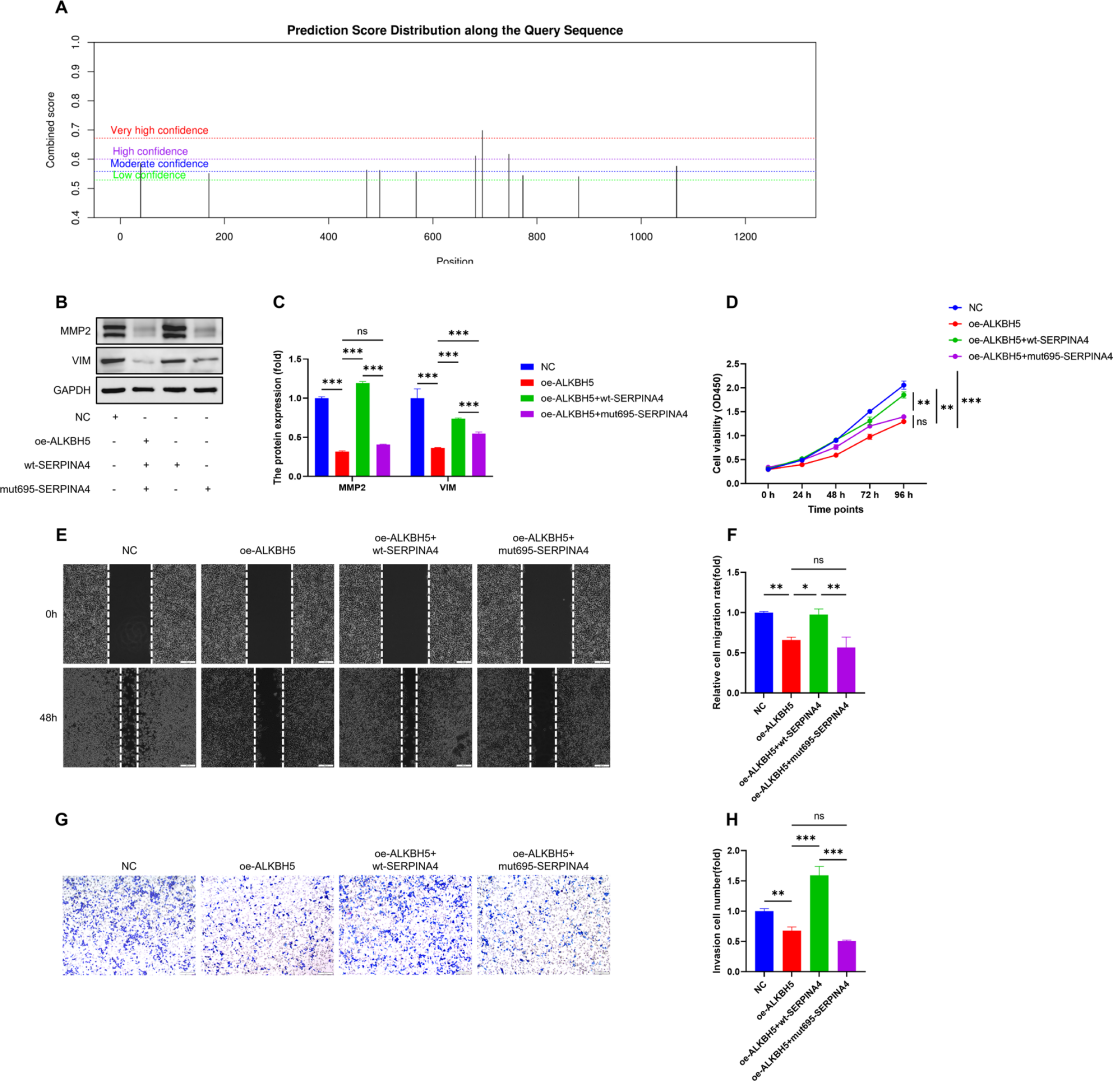
Rescue experiments validate m6A-dependency of ALKBH5–SERPINA4 signaling. (**A**) Schematic of WT SERPINA4 and the methylation-deficient mutant (mut695) designed based on SRAMP-predicted candidate m6A site(s) on SERPINA4 mRNA. (**B**–**C**) Western blot of MMP2 and VIM under rescue conditions. (**D**) CCK-8 proliferation assay. (**E**–**F**) Wound healing assay results. (**G**–**H**) Transwell invasion with WT vs. mutant SERPINA4. Data from three independent experiments; ANOVA with post hoc tests used. *, *P* < 0.05; **, *P* < 0.01, ***, *P* < 0.001

Given the emerging role of epitranscriptomic regulation in cancer, we further examined whether SERPINA4 expression is modulated by m⁶A modification and its associated regulators. To dissect the upstream regulatory mechanisms of SERPINA4, we investigated the role of the m6A demethylase ALKBH5. Overexpression of ALKBH5 noticeably decreased both the mRNA and protein levels of SERPINA4, whereas ALKBH5 knockdown led to a marked increase in SERPINA4 expression (Fig. 8A–D), indicating that ALKBH5 negatively regulates SERPINA4 in our CRC cell models. MeRIP analysis further demonstrated that ALKBH5 modulates the methylation status of SERPINA4 transcripts: ALKBH5 overexpression reduced m6A enrichment on SERPINA4 mRNA, while ALKBH5 silencing increased methylation (Fig. 8E–F). Consistently, actinomycin D chase assays showed that ALKBH5 overexpression shortened the half-life of SERPINA4 transcripts, supporting a role for ALKBH5 in reducing SERPINA4 mRNA stability (Fig. 7G–H).

RNA-binding protein IGF2BP3 was found to interact directly with SERPINA4 mRNA. RNA immunoprecipitation (RIP) assays revealed weaker SERPINA4–IGF2BP3 association in ALKBH5-overexpressing cells, while this interaction was enhanced in ALKBH5-silenced cells (Fig. 7I–J). Moreover, co-expression of IGF2BP3 further enhanced the stability of SERPINA4 transcripts compared to controls (Fig. 7K). These findings suggest that ALKBH5 modulates SERPINA4 mRNA stability through an m6A-associated mechanism and are consistent with the involvement of IGF2BP3; however, further loss-of-function studies are required to determine whether IGF2BP3 is essential for this regulation. Given that IGF2BP3 is a canonical m⁶A reader that preferentially stabilizes m⁶A-modified transcripts, the reduced SERPINA4–IGF2BP3 association upon ALKBH5 overexpression provides a mechanistic explanation for the decreased SERPINA4 mRNA stability.

### ALKBH5 suppresses CRC cell proliferation and metastasis via SERPINA4 in an m6A-dependent manner

To validate the functional relevance of ALKBH5–SERPINA4 signaling, and to test whether SERPINA4 mediates the tumor-suppressive effects of ALKBH5, we performed rescue assays using wild-type and mutant SERPINA4 constructs.To guide mutant design, candidate m6A sites on SERPINA4 mRNA were predicted using SRAMP, and the site around position 695 was selected because it showed the highest prediction confidence/combined score (Fig. 8A). ALKBH5 overexpression reduced the expression of MMP2 and VIM proteins, and re-expression of wild-type SERPINA4 restored these levels, whereas a methylation-deficient SERPINA4 mutant (mut695) failed to rescue (Fig. 8B–C). Cell proliferation assays revealed that ALKBH5 overexpression suppressed cell growth, and wild-type SERPINA4 rescued this inhibitory effect, whereas mut695 failed to rescue (Fig. 8D). Similarly, wound healing and transwell assays demonstrated that ALKBH5 overexpression inhibited migration and invasion, and wild-type SERPINA4 restored these phenotypes, whereas the mutant form failed to do so, but this restoration was lost when the mutant form was used (Fig. 8E–H). Altogether, these data support an m6A-dependent functional link between ALKBH5 and SERPINA4 in CRC cells; further studies are needed to validate the ALKBH5–SERPINA4 axis in vivo and to test the necessity of IGF2BP3.

## Discussion

Our study supports an oncogenic role of SERPINA4 in CRC and further provides mechanistic insight into its epitranscriptomic regulation. Through integrative analysis of large-scale datasets and experimental validation, we identified that high SERPINA4 expression is consistently associated with poor clinical outcomes, including reduced overall survival and disease-specific endpoints. This prognostic value was further reinforced by the development of a nomogram integrating SERPINA4 with clinical parameters, which yielded robust survival predictions [37].

Functionally, SERPINA4 exerts a pro-oncogenic influence by promoting CRC cell proliferation, migration, and invasion both in vitro and in vivo. At the molecular level, SERPINA4 overexpression was associated with increased levels of key mesenchymal markers such as MMP2 and VIM, consistent with an invasive/EMT-like phenotype. The oncogenic potential of SERPINA4 was further substantiated in xenograft models, where its overexpression significantly enhanced tumor growth and altered the expression of angiogenic and invasive markers [6, 38].

As a canonical serpin, SERPINA4 is best known for inhibiting tissue kallikrein and related serine proteases. Although this protease-inhibitory activity could plausibly contribute to invasion-associated processes (e.g., protease-dependent extracellular matrix remodeling), the present study did not directly measure protease activity or employ protease-inhibitory–defective mutants to distinguish protease-dependent versus protease-independent mechanisms in CRC. Therefore, the EMT-related marker changes reported here should be interpreted as phenotype-associated readouts rather than definitive evidence for a protease-inhibition-driven mechanism.

In addition, m6A regulation is mediated by a coordinated network of writers, erasers, and readers. While our data support an ALKBH5-dependent change in m6A enrichment on SERPINA4 and an IGF2BP3-associated stabilization mechanism, we did not systematically evaluate other demethylases (e.g., FTO) or additional reader proteins (e.g., YTHDF1/2) that may influence SERPINA4 turnover and/or translation. Thus, we cannot exclude parallel or context-specific regulation of SERPINA4 by other m6A factors, which merits further investigation.Further studies will also be important to clarify the mechanistic involvement of IGF2BP3 in ALKBH5-associated regulation of SERPINA4 and to validate the ALKBH5–SERPINA4 axis in vivo. In addition, although mut695 was prioritized based on SRAMP prediction as a high-confidence candidate site, additional potential m⁶A sites on SERPINA4 mRNA may exist and should be systematically mapped and evaluated in future work (e.g., m⁶A-seq).

At the molecular level, we uncovered that SERPINA4 expression is regulated post-transcriptionally through an m6A-dependent pathway. This epitranscriptomic regulatory layer offers an upstream explanation for SERPINA4 dysregulation in CRC, which we further delineated by focusing on ALKBH5-mediated m⁶A demethylation and IGF2BP3-dependent transcript stabilization. Specifically, ALKBH5 decreases SERPINA4 mRNA stability by removing m⁶A marks on SERPINA4 transcripts, thereby reducing IGF2BP3 binding and attenuating m⁶A-dependent stabilization. This interpretation is in line with the established m⁶A-reader function of IGF2BP3, which typically stabilizes transcripts through preferential binding to m⁶A-modified RNA. Rescue experiments using methylation-deficient constructs confirmed that the tumor-suppressive effects of ALKBH5 are contingent upon intact m6A modification sites within SERPINA4 [39–41].

Importantly, our analyses suggest a potential association between SERPINA4 expression and the tumor immune microenvironment. Correlation analyses revealed links with immune-modulatory genes, including immune checkpoints, chemokines, MHC molecules, and antigen receptor components. SERPINA4’s association with reduced CD8 + T and NK cell infiltration should be interpreted as a correlative observation [42, 43]. Further experimental studies may help clarify whether SERPINA4 plays a direct role in tumor–immune interactions, which we view as an important direction for future work.

## Conclusion

In conclusion, SERPINA4 is a multifaceted gene associated with malignant behavior and post-transcriptional regulatory dynamics in CRC, and its relationship with immune microenvironment features warrants further investigation. These findings establish SERPINA4 not only as a robust prognostic indicator but also as a promising therapeutic target for precision oncology.

Clinically, integrating SERPINA4 expression profiling may refine current risk stratification frameworks and enhance the predictive performance of nomograms beyond traditional histopathological metrics. Furthermore, given its role in immune modulation, SERPINA4 could serve as a biomarker for patient selection in immunotherapy trials or as a combinatorial target in immune-based regimens.

Mechanistically, the ALKBH5–m6A–IGF2BP3 regulatory axis enriches our understanding of epitranscriptomic regulation in CRC. This pathway underscores how dynamic RNA modifications can drive cancer progression and may offer novel avenues for therapeutic intervention focused on RNA methylation machinery [39, 40].

Altogether, our results provide compelling preclinical evidence that targeting the ALKBH5–SERPINA4 axis could serve as a dual-approach strategy to inhibit both tumor cell intrinsic growth and immune escape, for example, by restoring ALKBH5 activity or inhibiting SERPINA4. Future clinical studies and drug development pipelines should explore inhibitors or modulators of this pathway for their translational potential.

## Supplementary Information

Below is the link to the electronic supplementary material.

Supplementary Material 1

## Data Availability

The datasets supporting the conclusions of this article are available in the GEO database, with the accession number GSE14333 (https://www.ncbi.nlm.nih.gov/geo/query/acc.cgi?acc=GSE14333). TCGA-COAD bulk RNA sequencing (RNA-seq) data and survival information are available in the UCSC Xena browser (https://xenabrowser.net/datapages/).
