# Supplementary material for: SERPINA4 facilitates colorectal cancer progression through m⁶A-dependent stabilization
Source: J Transl Med. 2026 Feb 28;24:474. doi: 10.1186/s12967-026-07810-1 (PMC13059541; doi:10.1186/s12967-026-07810-1)

Figure 5D

original

maker merged

SERPINA4

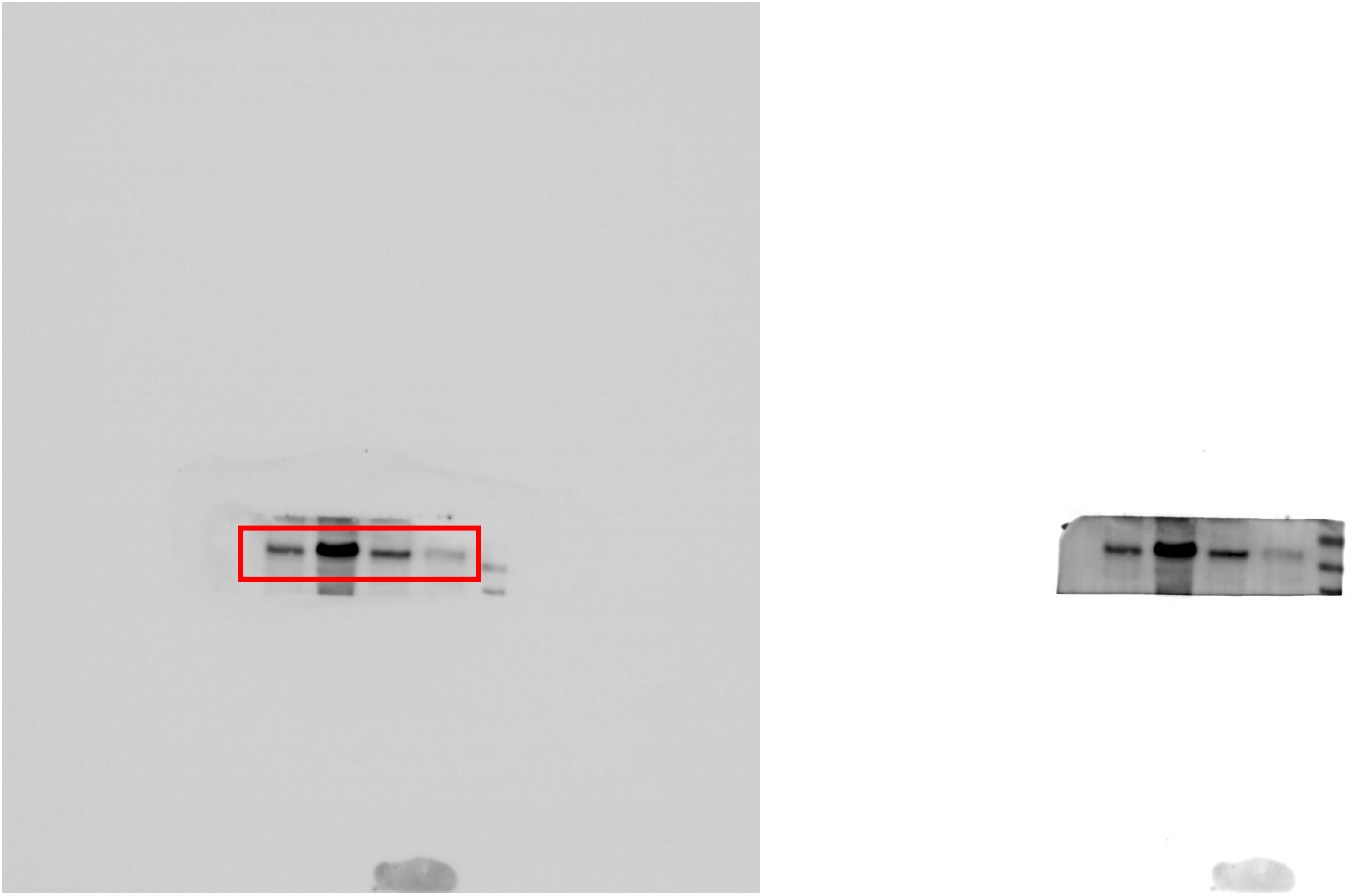

Figure 5D

original

maker merged

MMP2

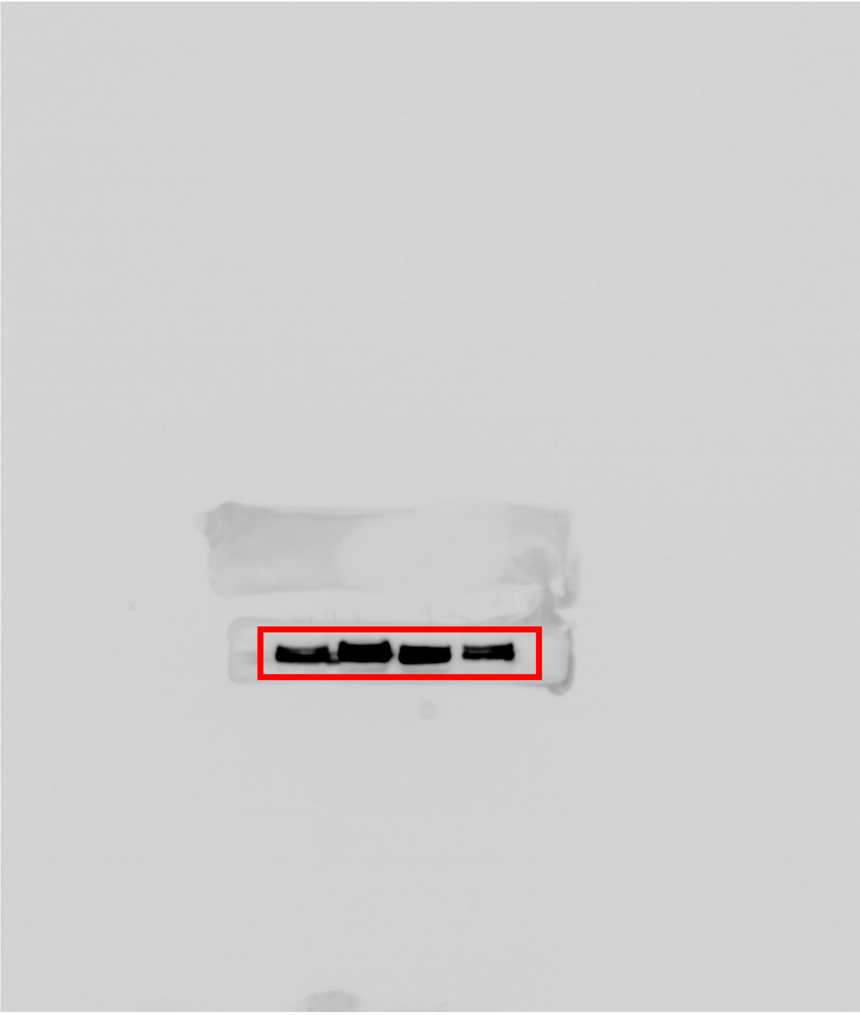

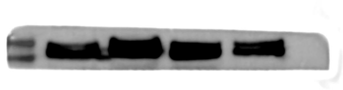

Figure 5D

original

maker merged

VIM

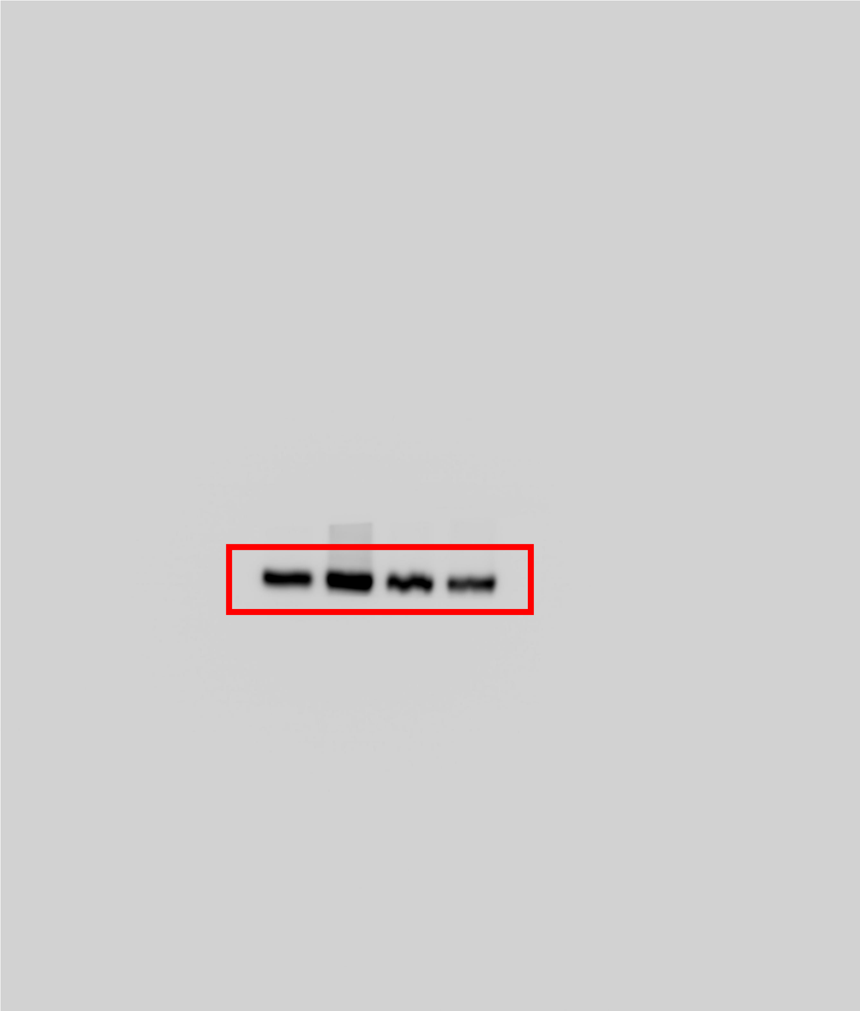

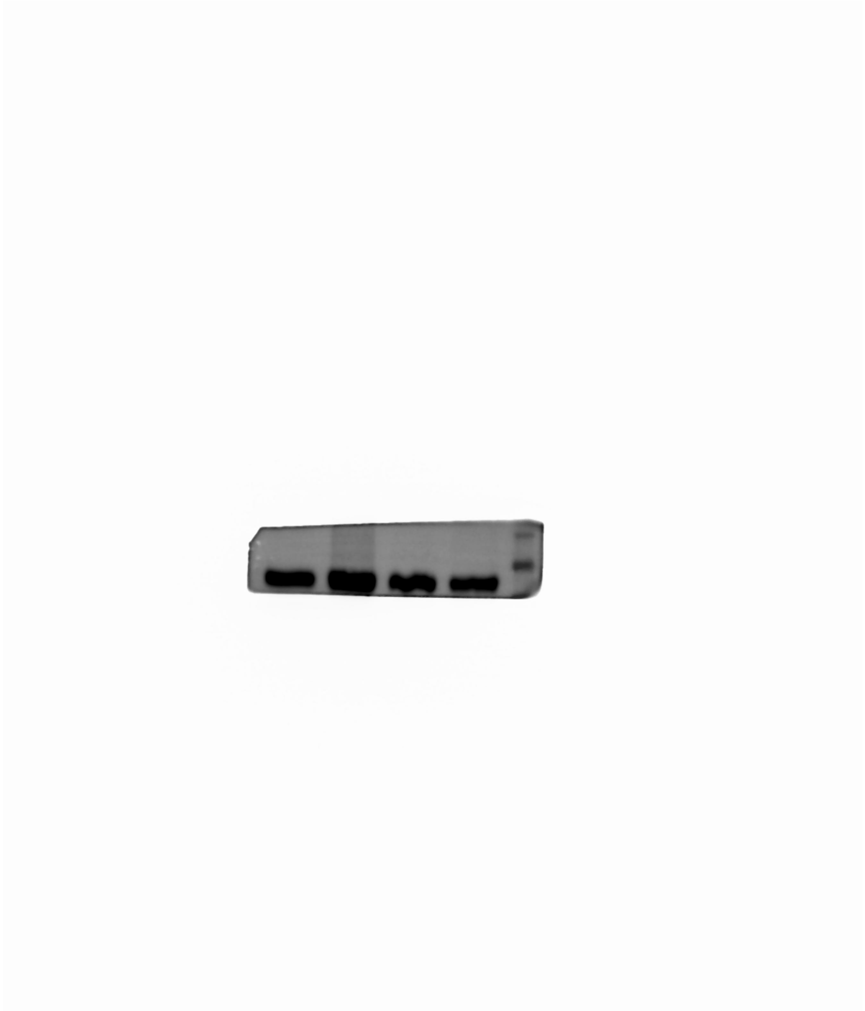

Figure 5D

original

maker merged

GAPDH

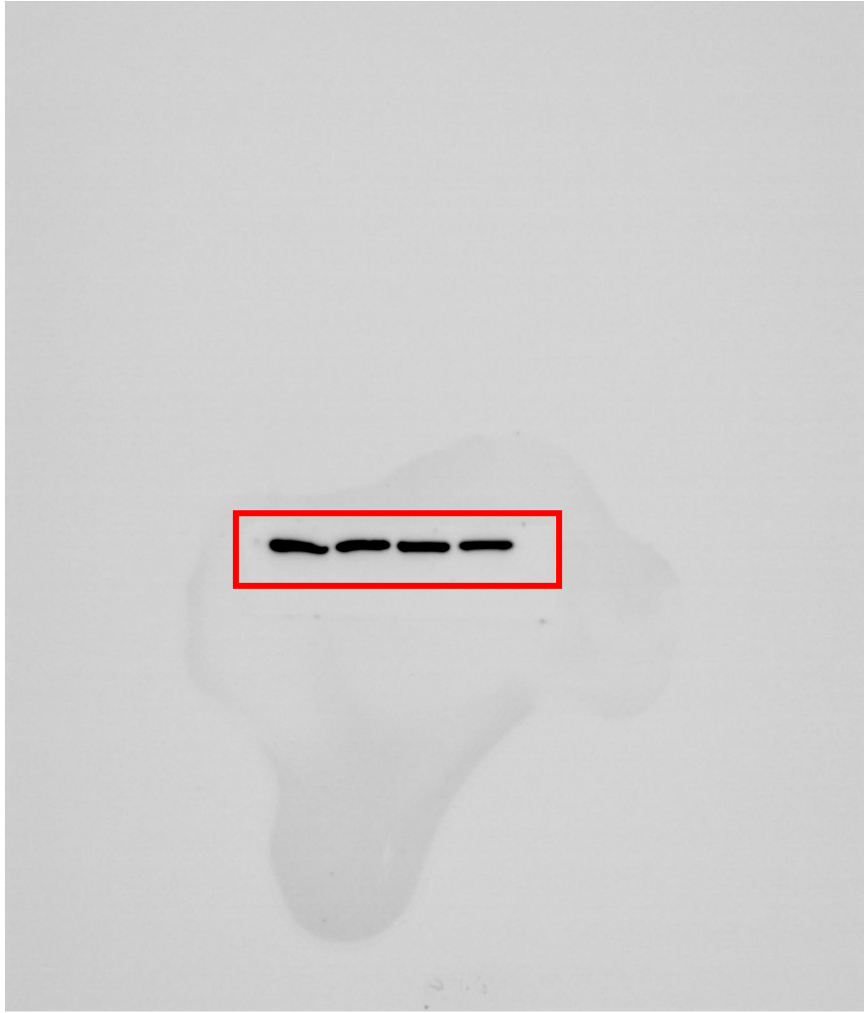

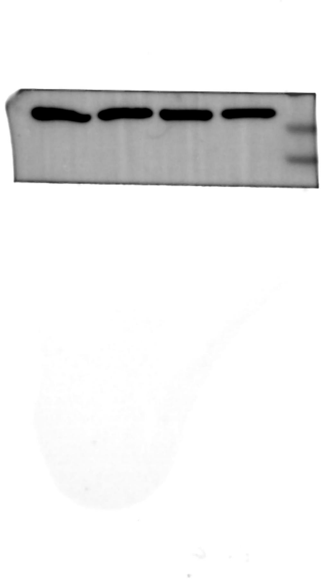

Figure 6H

original

maker merged

SERPINA4

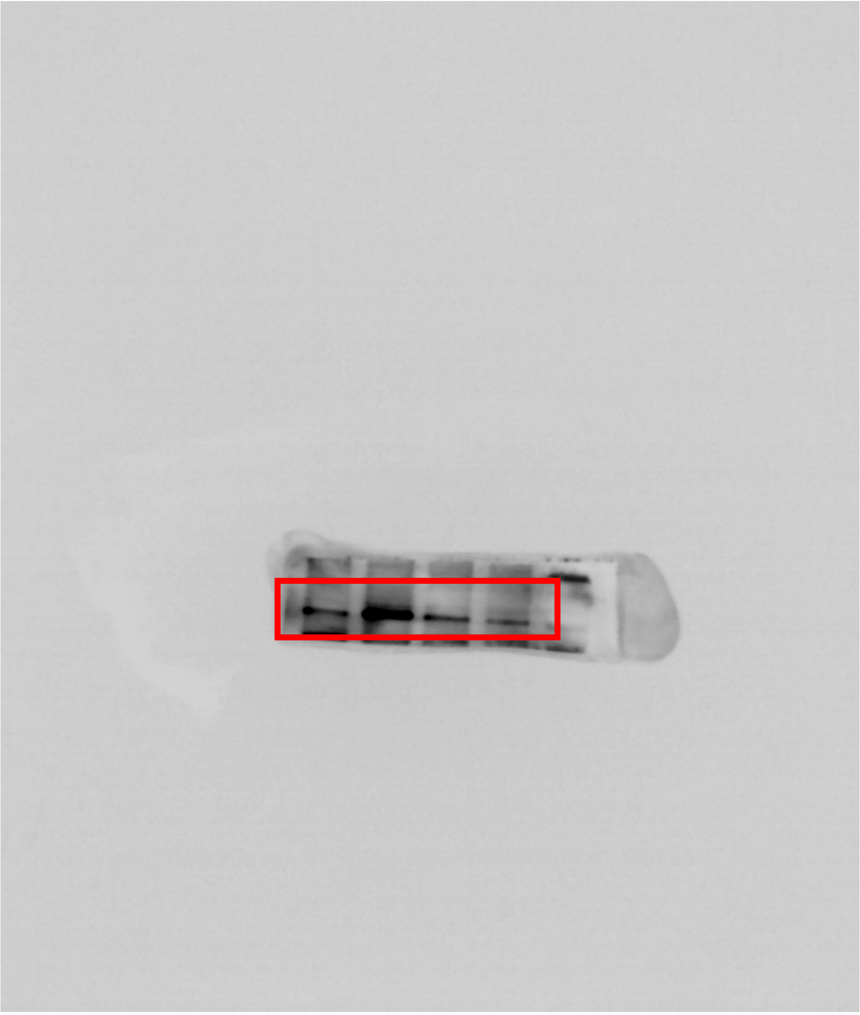

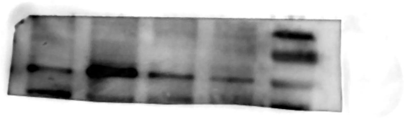

Figure 6H

original

maker merged

GAPDH

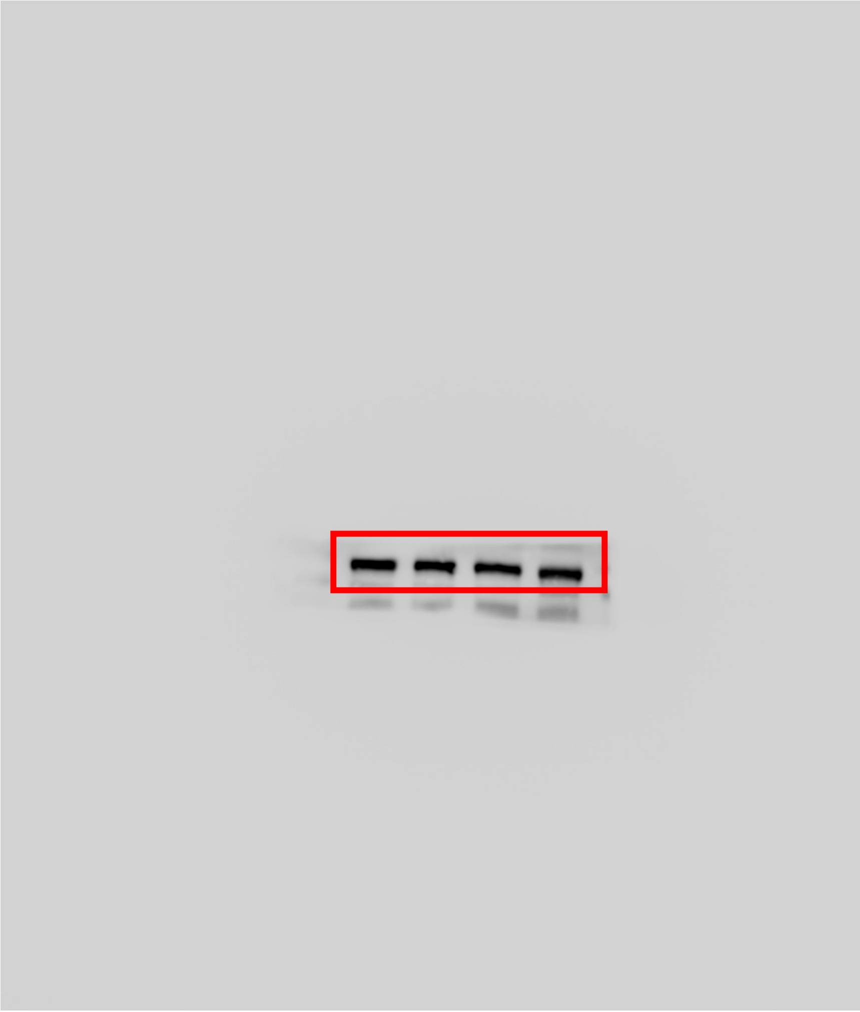

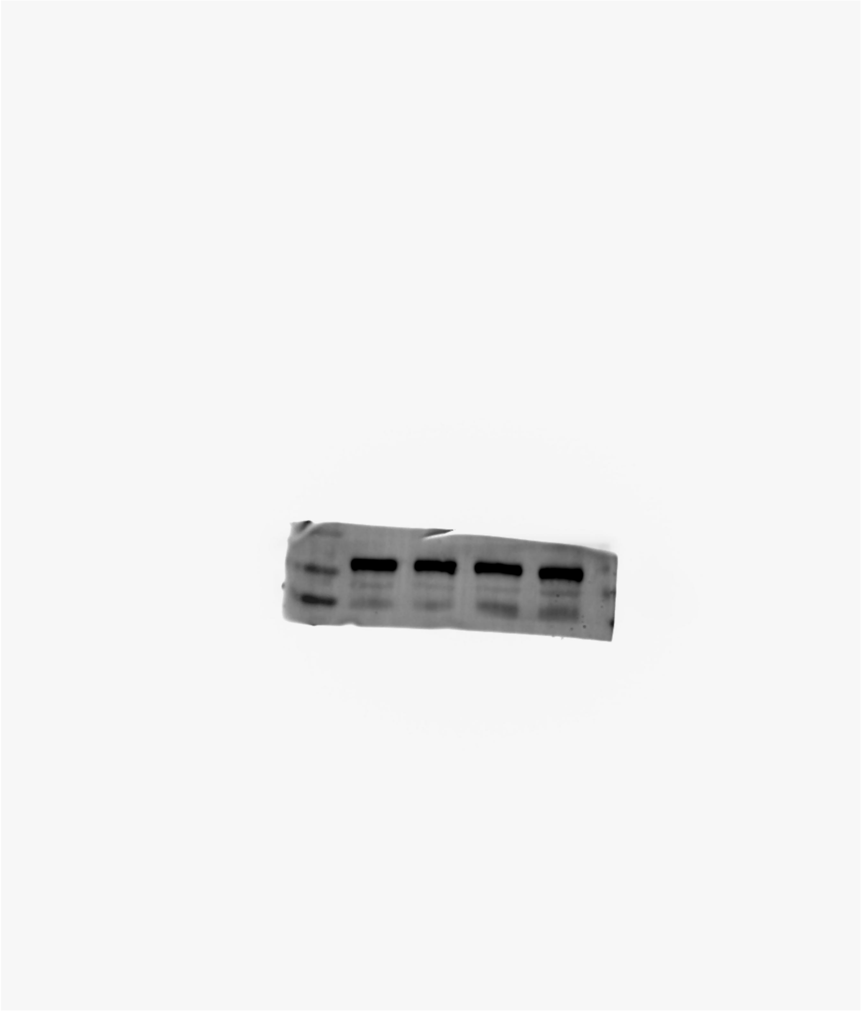

Figure 7C

original

maker merged

SERPINA4

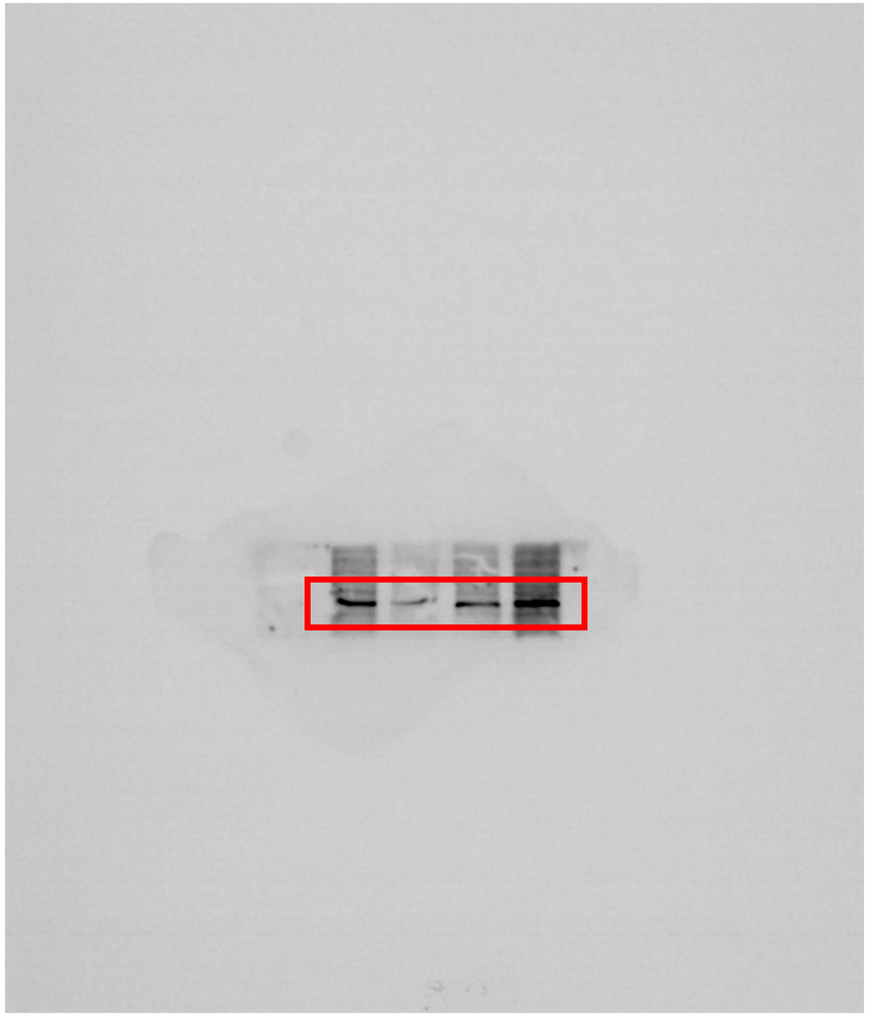

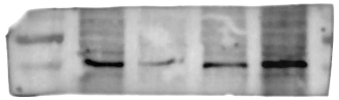

Figure 7C

original

maker merged

GAPDH

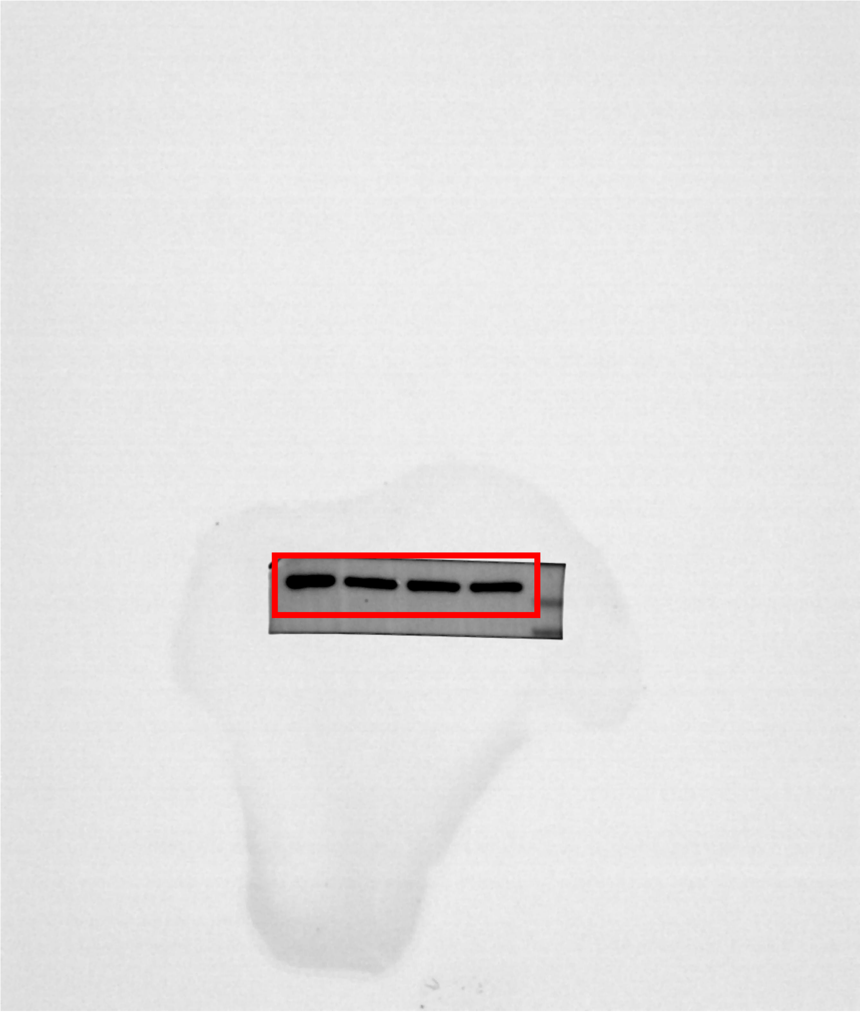

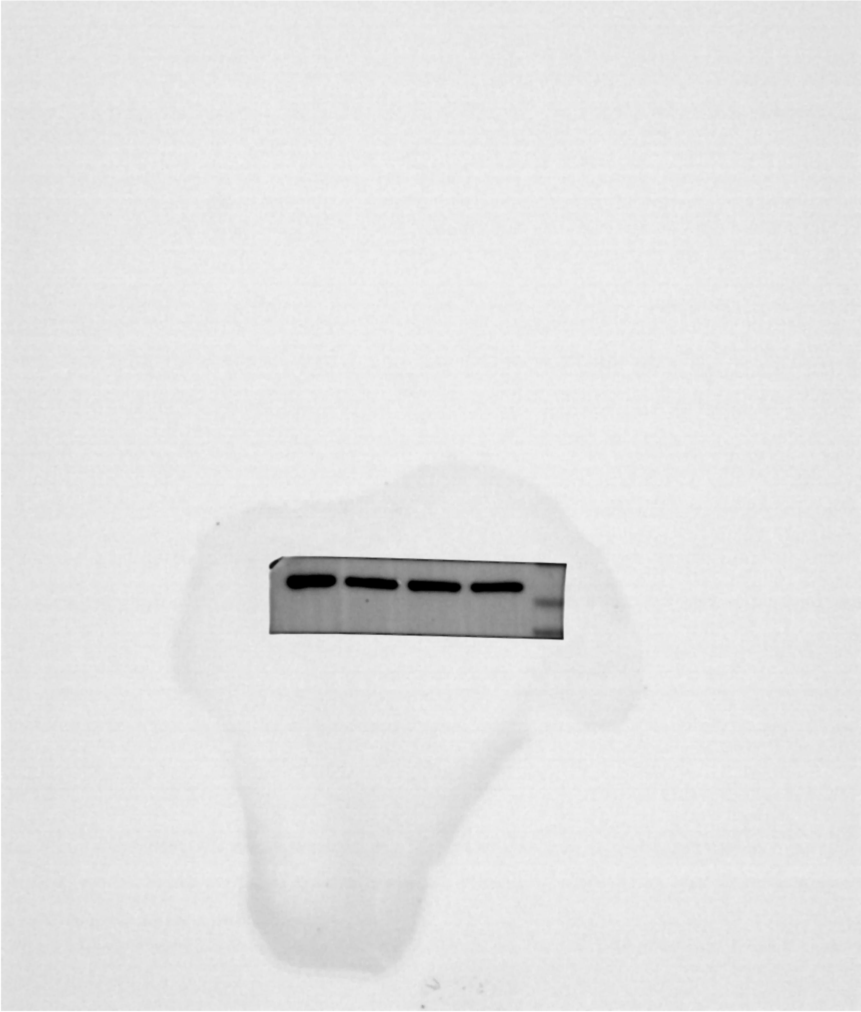

Figure 8B

original

maker merged

MMP2

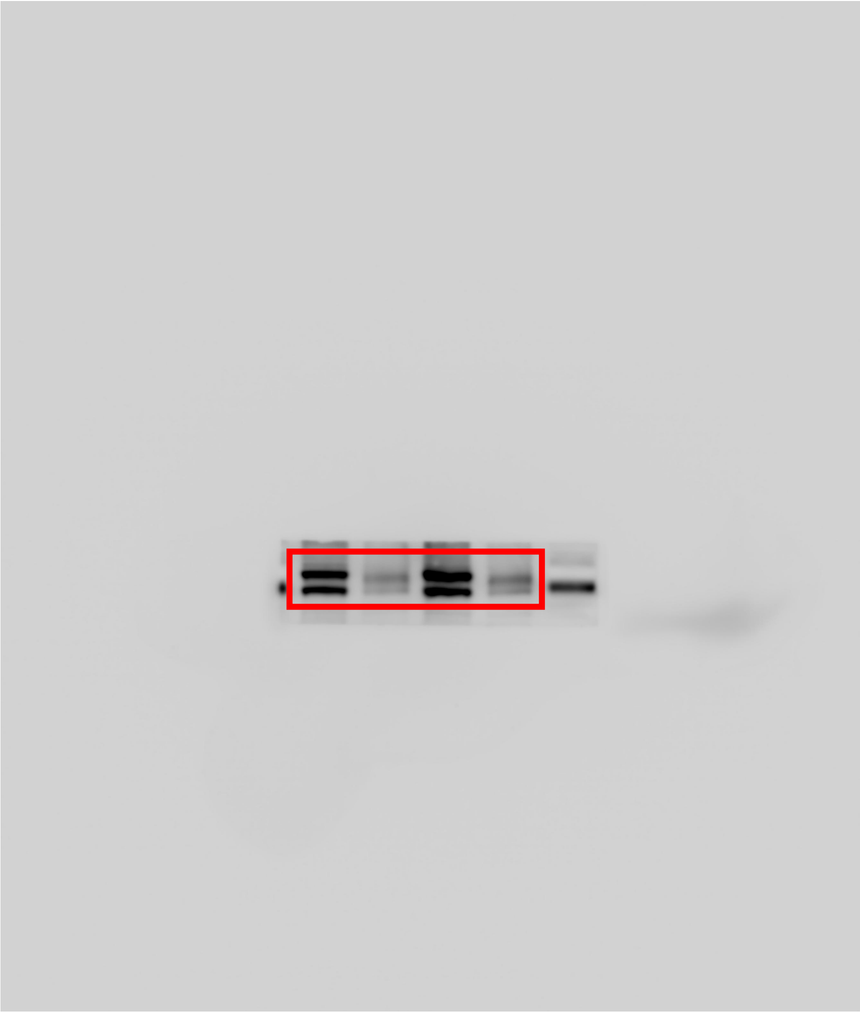

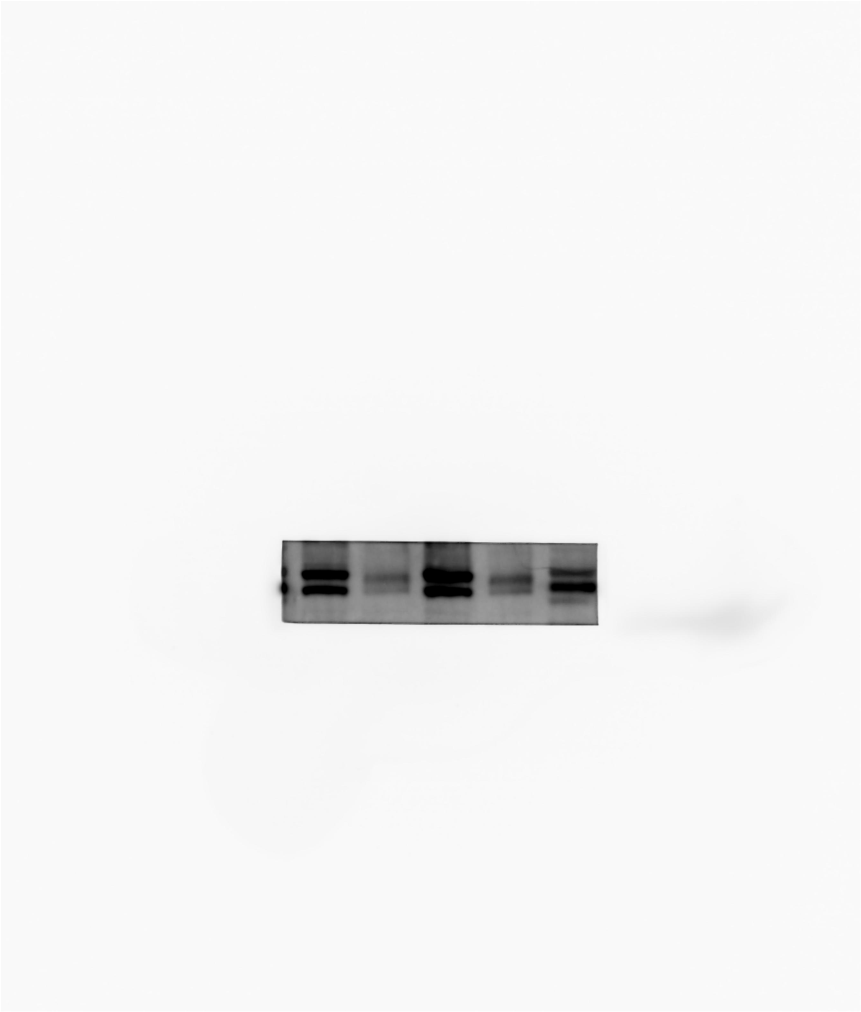

Figure 8B

original

maker merged

VIM

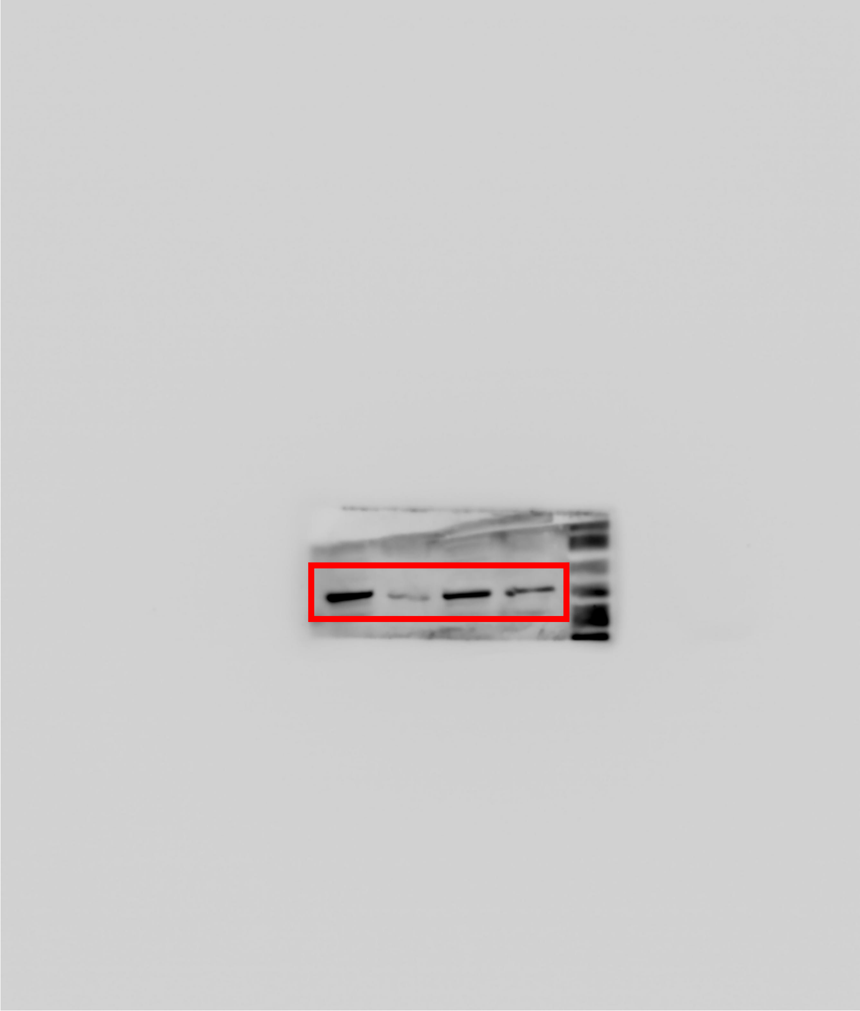

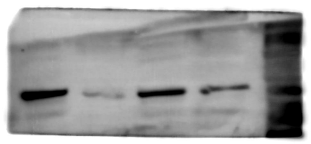

Figure 8B

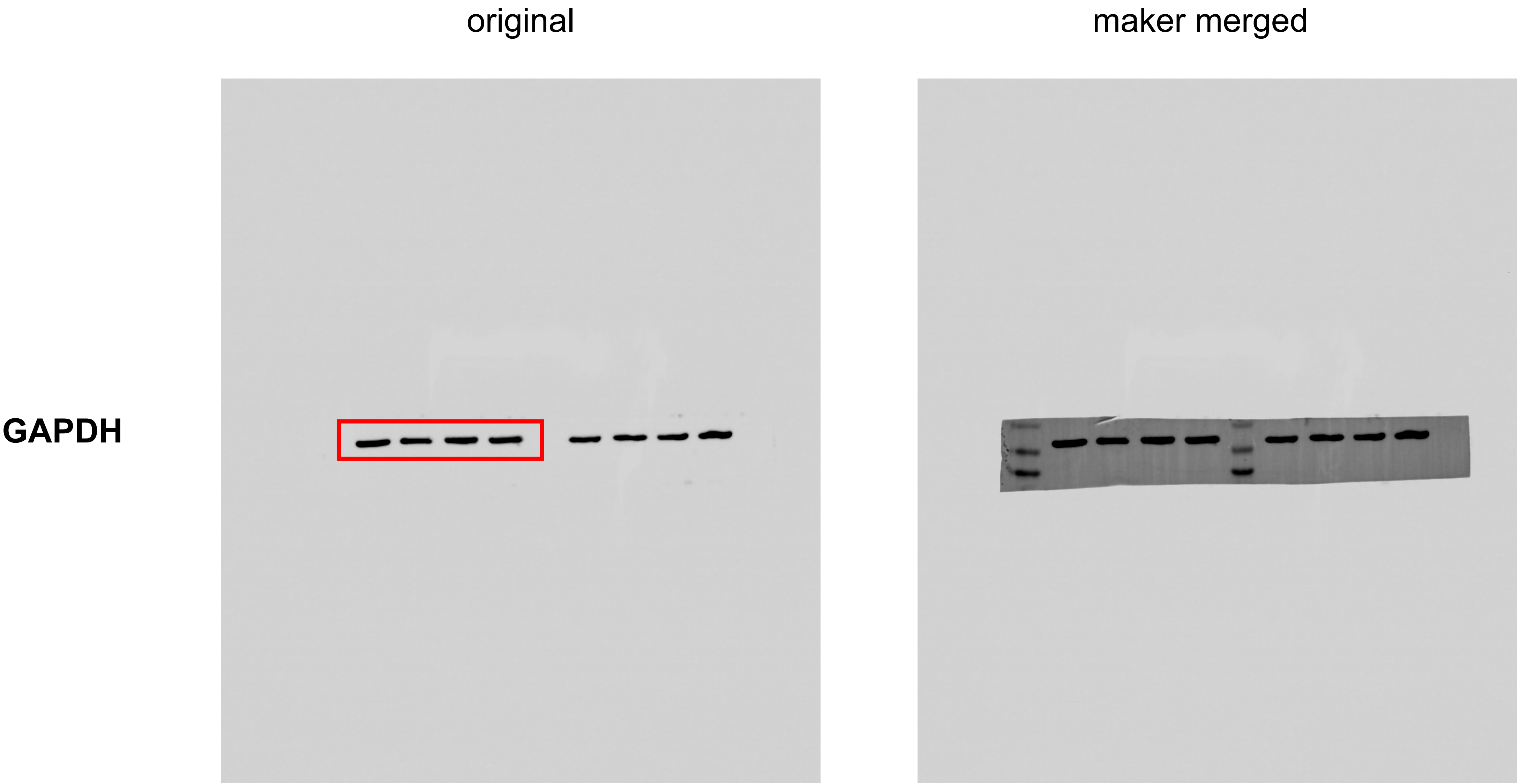

Supplement: Supplementary file 1 — Supplementary Material 1 [file 12967_2026_7810_MOESM1_ESM.pdf]
